# Supplementary material for: Does plasmid-based beta-lactam resistance increase E. coli infections: Modelling addition and replacement mechanisms
Source: PLoS Comput Biol. 2022 Mar 14;18(3):e1009875. doi: 10.1371/journal.pcbi.1009875 (PMC8947615; doi:10.1371/journal.pcbi.1009875)

**S1 Text. Differential equations of the mathematical model.**
The parameters used in the model can be found in S4 Table. The differential equations for the three sub-populations in total are:


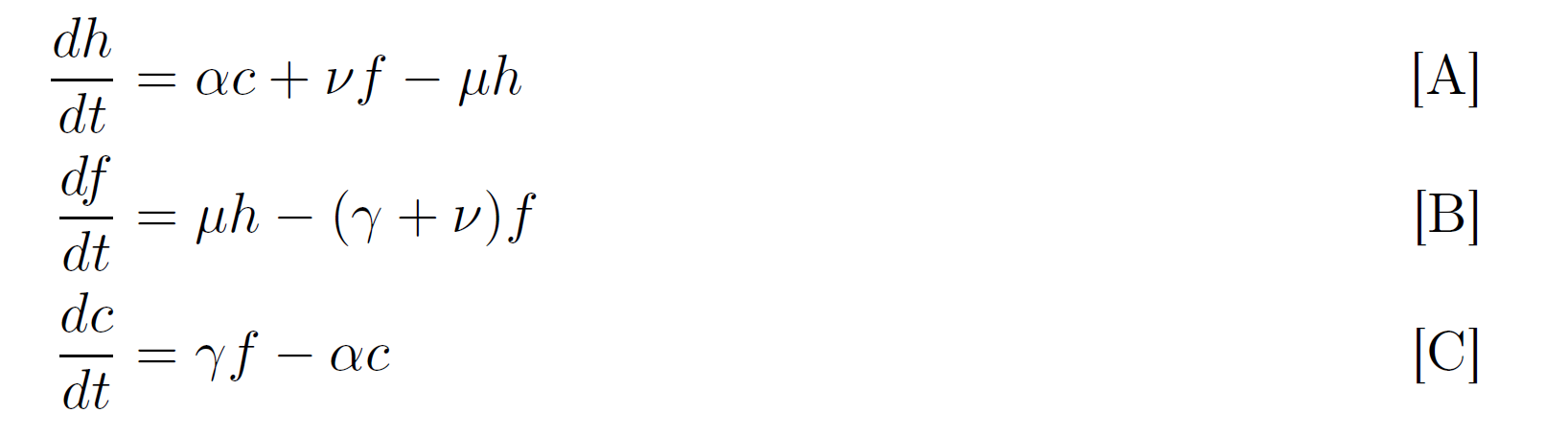


The differential equations for the hospital population are:


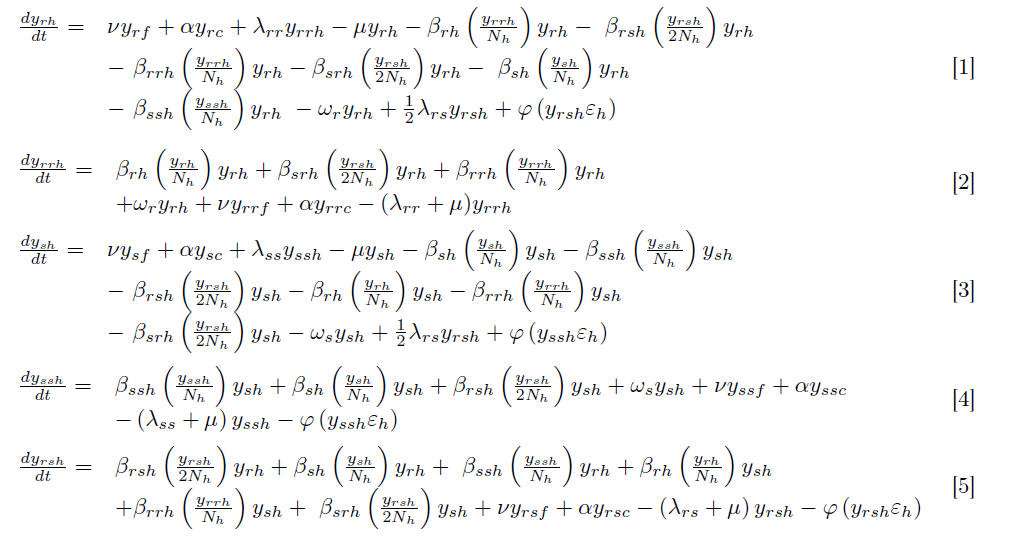


The differential equations for the former patient population are:


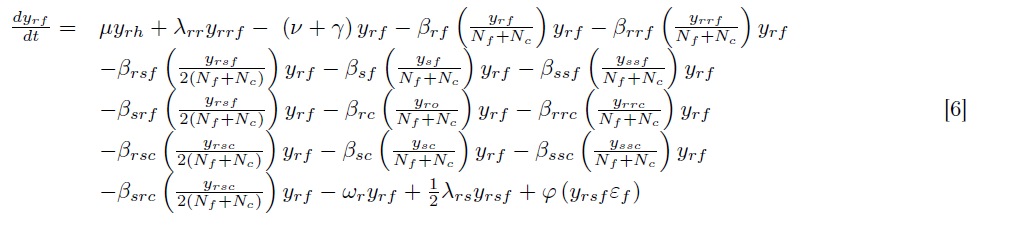


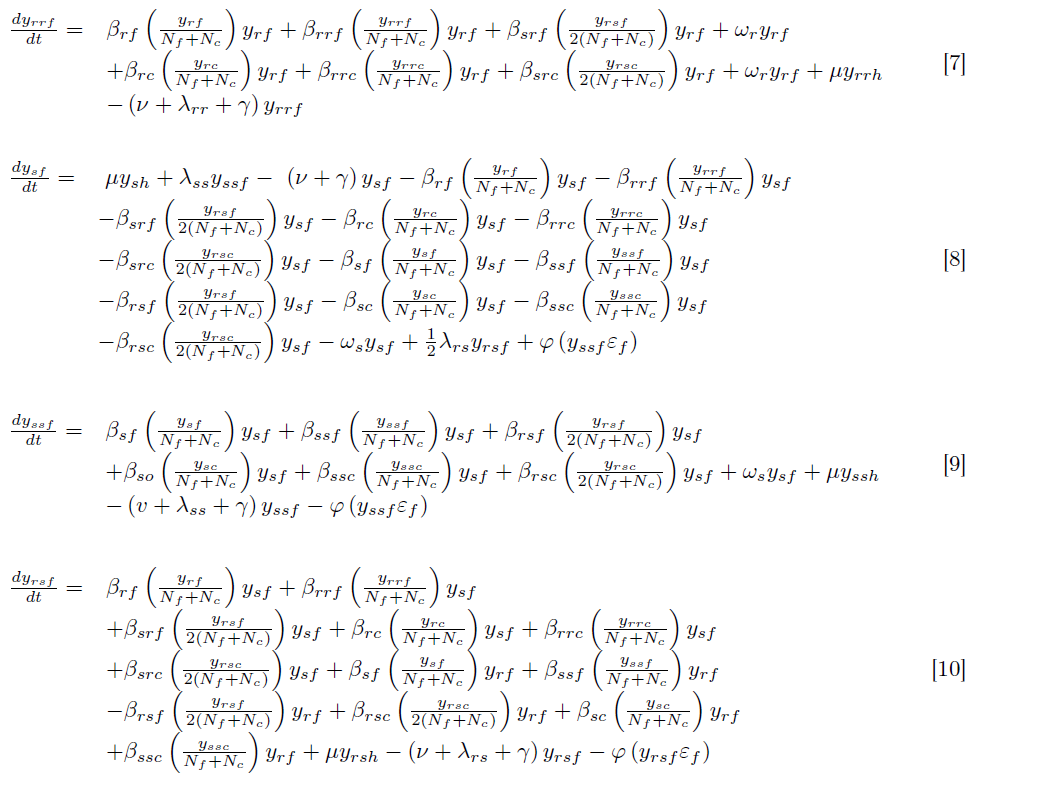


The differential equations for the community are:


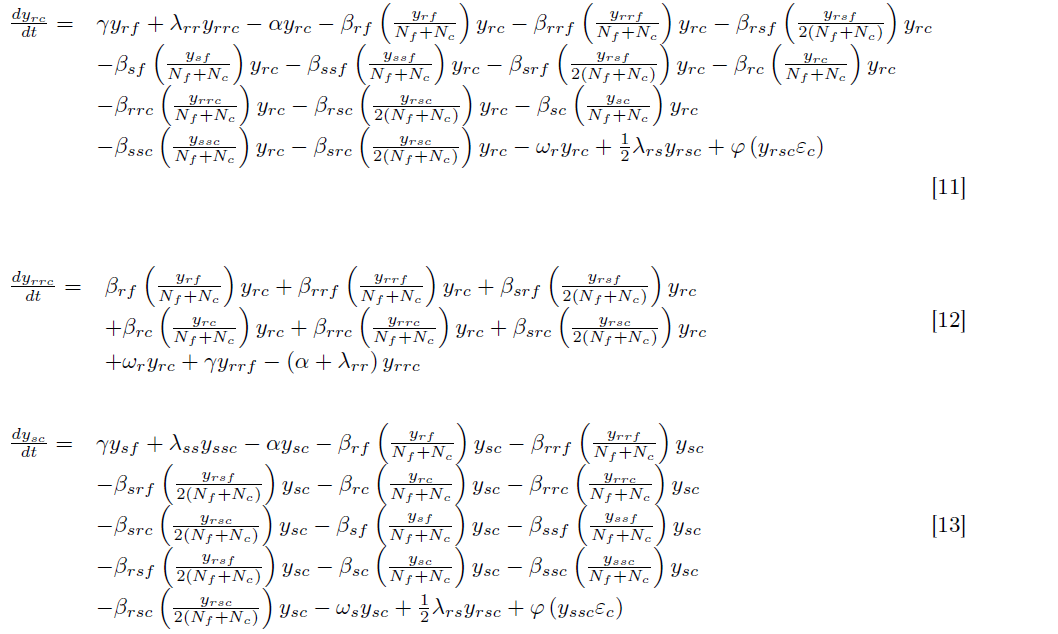


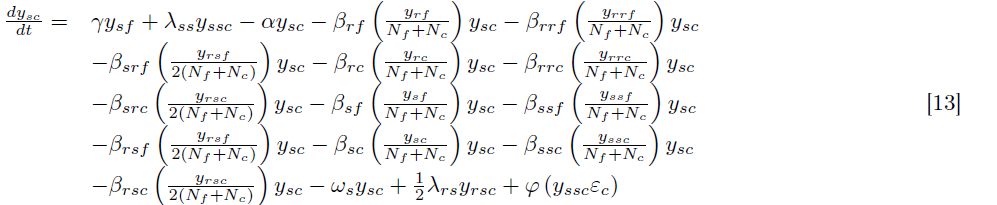

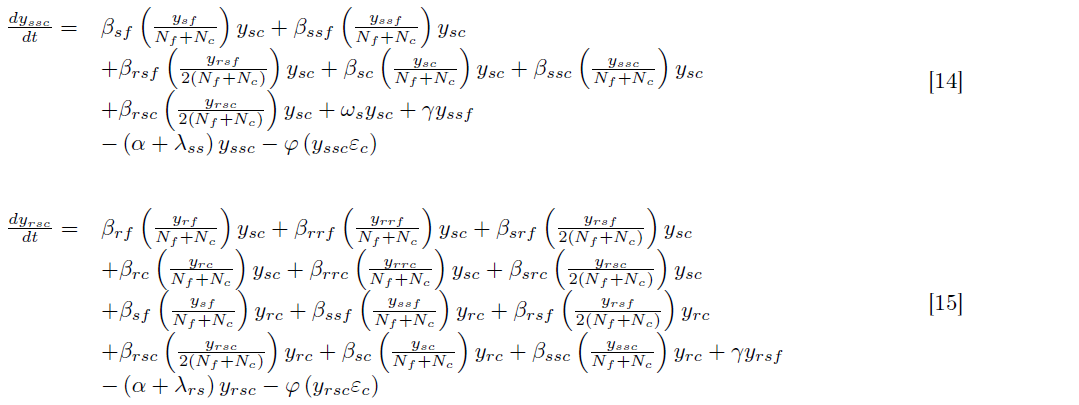


The equations to calculate the number of infections are:

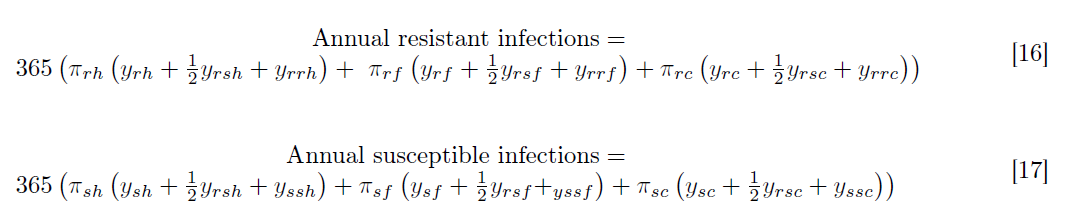

Supplement: S1 Text — (DOCX) [file pcbi.1009875.s001.docx]
