# Supplementary material for: Does plasmid-based beta-lactam resistance increase E. coli infections: Modelling addition and replacement mechanisms
Source: PLoS Comput Biol. 2022 Mar 14;18(3):e1009875. doi: 10.1371/journal.pcbi.1009875 (PMC8947615; doi:10.1371/journal.pcbi.1009875)
Supplement: S2 Text — (DOCX) [file pcbi.1009875.s002.docx]

**S2 Text. Infection rate calculations**

In 2017, 159,619 infections with *E. Coli* were cultured in 34 laboratories the Netherlands [1]. Of these, 57% occurred at the general practitioner and 15% in outpatients. Thus, in total roughly 72% of the *E. coli* infections that were severe enough to pose the need to be cultured in 2017 occurred in the community, which are 114,925 infections.
 The Netherlands had 17,08 million inhabitants in 2017. In our model, 0.177% of the people is in the hospital, Thus, this would mean that in 2017 (1-0.00177)*17.08= 17.05 people were in the community. Based on the number of infections in de Greeff & Mouton, 2018 [1], the infection rate per year per person is 114,925/170,497684 =6.7406x10^-4^ and the infection rate per day per person is 6.7406x10^-4^/365=1.846745046839801x10^-6^‬. However, the number of infections reported in de Greeff & Mouton [1] is an underrepresentation of all infections in the Netherlands, as not all Dutch medical microbiological laboratories were included in the analyses of this report.
 To arrive at a total of 72% of the infections occurring in the community (as observed in the Netherlands), we have to adjust this rate. Based on the previously research transmission rates we reported in S2 Table, 684 infections would occur in the hospital. If this is 28% of the total number of infections, then 1,758 infections should occur in the community. As 1,342 infections occur in the former patient population based on the estimates in S2 Table, 416 should occur in the community. With a rate of 1.846745046839801x10^-6,^ only 66 infections would occur in the community which is as expected an underestimate. Dividing 416 by 66 gives 6.22589616612008, and multiplying the infection rate of the community with this number gives a total number of 416 infections in the community. To arrive at this number, we have to multiply the infection rate of 1.846745046839801x10^-6^‬ with 6.22589616612008.

**References**

1. de Greeff SC, Mouton JW. NethMap 2018: Consumption of antimicrobial agents and antimicrobial resistance among medically important bacteria in the Netherlands / MARAN 2018: Monitoring of Antimicrobial Resistance and Antibiotic Usage in Animals in the Netherlands in 2017 [Internet]. Rijksinstituut voor Volksgezondheid en Milieu RIVM; 2018. Available from: https://rivm.openrepository.com/bitstream/10029/622042/2/2018-0046.pdf
