# Supplementary material for: Does plasmid-based beta-lactam resistance increase E. coli infections: Modelling addition and replacement mechanisms
Source: PLoS Comput Biol. 2022 Mar 14;18(3):e1009875. doi: 10.1371/journal.pcbi.1009875 (PMC8947615; doi:10.1371/journal.pcbi.1009875)
Supplement: S3 Text — (DOCX) [file pcbi.1009875.s003.docx]

**S3 Text. Calculations admission and readmission rates**

In 2017, the Netherlands had a total number of 37,753 hospital beds [1]. Assuming an occupancy of 80%, this would mean that per day there were 0.8*37,753 = 30,202 people in the hospital. Since there were 17,08 million inhabitants in 2017, a proportion of 30,202/17,0800000 = 0.00177 of the Dutch population is in the hospital. Per 100,000 inhabitants this is 177 people per day that stay in the hospital.

To arrive at this number, the estimates of Cooper et al. [2] have to be multiplied with a fraction of 0.333183. We arrived at this number by solving equation A-C, given that the hospital population is 177. The admission and readmission rate will stay proportional to the estimates in Cooper et al. [2] and now become 0.00063*0.333183 = 0.00020990529 and 0.0057*0.333183 = 0.0018991431, which indeed results in 177 people in the hospital per day per 100,000.

**References**

1. Deuning C. Aantal ziekehuisbedden [Internet]. 2019 [cited 2019 Dec 12]. Available from: https://www.volksgezondheidenzorg.info/onderwerp/ziekenhuiszorg/cijfers-context/aanbod#node-aantal-ziekenhuisbedden

2. Cooper BS, Medley GF, Stone SP, Kibbler CC, Cookson BD, Roberts JA, et al. Methicillin-resistant Staphylococcus aureus in hospitals and the community: stealth dynamics and control catastrophes. Proc Natl Acad Sci U S A. 2004 Jul;101(27):10223–8.
