# Supplementary material for: Does plasmid-based beta-lactam resistance increase E. coli infections: Modelling addition and replacement mechanisms
Source: PLoS Comput Biol. 2022 Mar 14;18(3):e1009875. doi: 10.1371/journal.pcbi.1009875 (PMC8947615; doi:10.1371/journal.pcbi.1009875)
Supplement: S4 Text — (DOCX) [file pcbi.1009875.s004.docx]

**S4 Text. External plasmid transfer**

Plasmid transfer may occur from other resistant pathogens to *E. coli.* In this mechanism, we model plasmid transfer from other sources. We add a route from SS to SR and from SS to RR. In addition, we assume that when an individual colonized with just an S strain acquires a resistant plasmid, the individual moves to SR rather than R because not all susceptible *E. coli* the person is colonized with will become resistant immediately. This results in the compartmental model in S1 Fig.

We started with a rate of 1% and increased this rate with 10% each time, thus ending with a rate of 2% (a 100% increase of 1%). We also modelled a rate 0·1% and increased it with 10%, ending with a rate of 0.2%. Both scenarios caused for total replacement of the susceptible strain after 50 years.
