# Supplementary material for: Does plasmid-based beta-lactam resistance increase E. coli infections: Modelling addition and replacement mechanisms
Source: PLoS Comput Biol. 2022 Mar 14;18(3):e1009875. doi: 10.1371/journal.pcbi.1009875 (PMC8947615; doi:10.1371/journal.pcbi.1009875)
Supplement: S5 Text — (DOCX) [file pcbi.1009875.s005.docx]

**S5 Text. Calculations of increased virulence**

S8 Table shows the observed incidence density of Enterobacteriaceae in the paper of Ammerlaan et al. [1] and the expected incidence density of Enterobacteriaceae if the ARB Enterobacteriaceae would have grown equally.

An increase of virulence of 10.5 times is needed to arrive at 10·12 instead of 0.97. This is estimated as 10.2 / 0.97.

**References**

1. Ammerlaan HSM, Harbarth S, Buiting AGM, Crook DW, Fitzpatrick F, Hanberger H, et al. Secular trends in nosocomial bloodstream infections: antibiotic-resistant bacteria increase the total burden of infection. Clin Infect Dis. 2013 Mar;56(6):798–805.
