# Supplementary figures and images for: Does plasmid-based beta-lactam resistance increase E. coli infections: Modelling addition and replacement mechanisms
Source: PLoS Comput Biol. 2022 Mar 14;18(3):e1009875. doi: 10.1371/journal.pcbi.1009875 (PMC8947615; doi:10.1371/journal.pcbi.1009875)

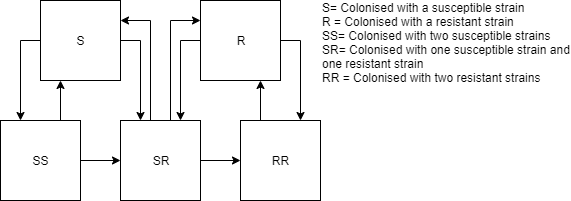


S1 Fig. Compartmental model of E. coli with plasmid transfer from other sources

Supplement: S1 Fig — (DOCX) [file pcbi.1009875.s006.docx]

**
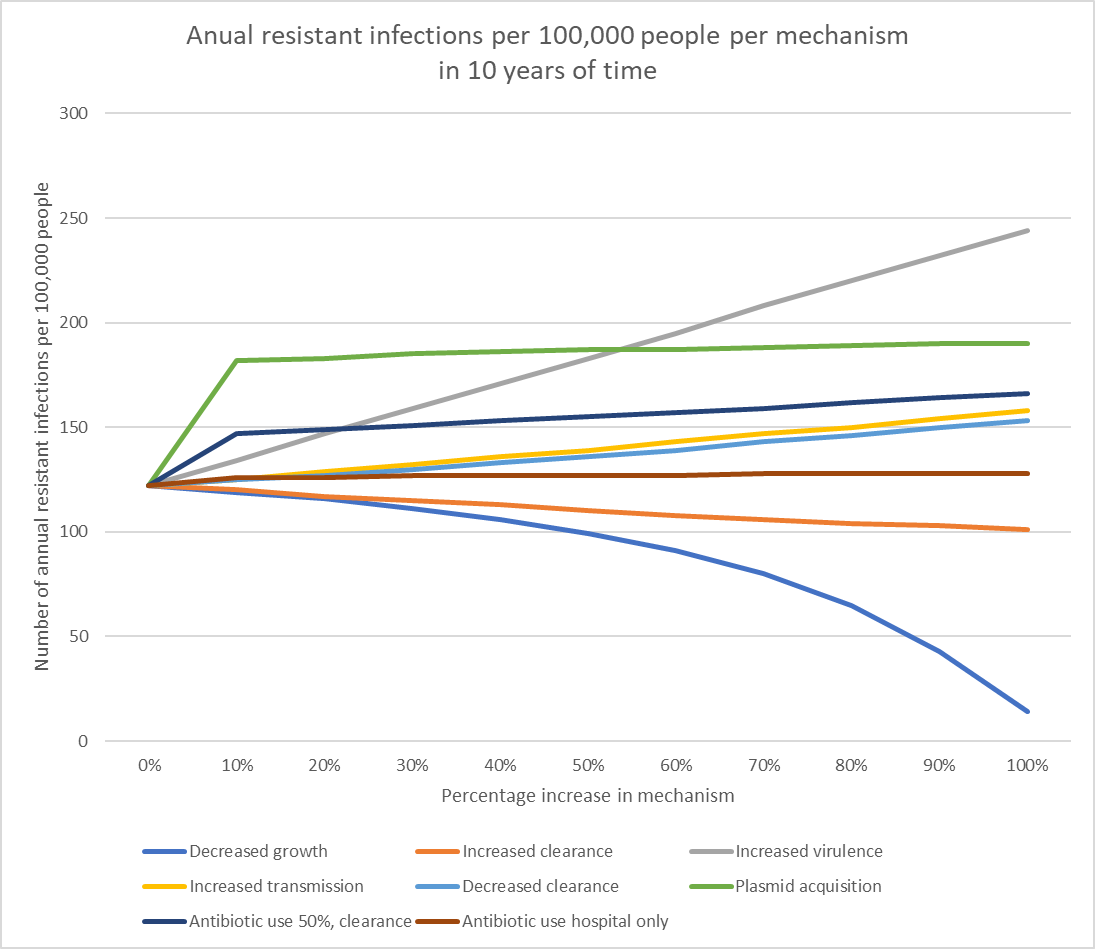
**

S2 Fig. Annual ESBL *E. coli* infections per 100,000 people per mechanism in 10 years of time

Supplement: S2 Fig — (DOCX) [file pcbi.1009875.s007.docx]

*
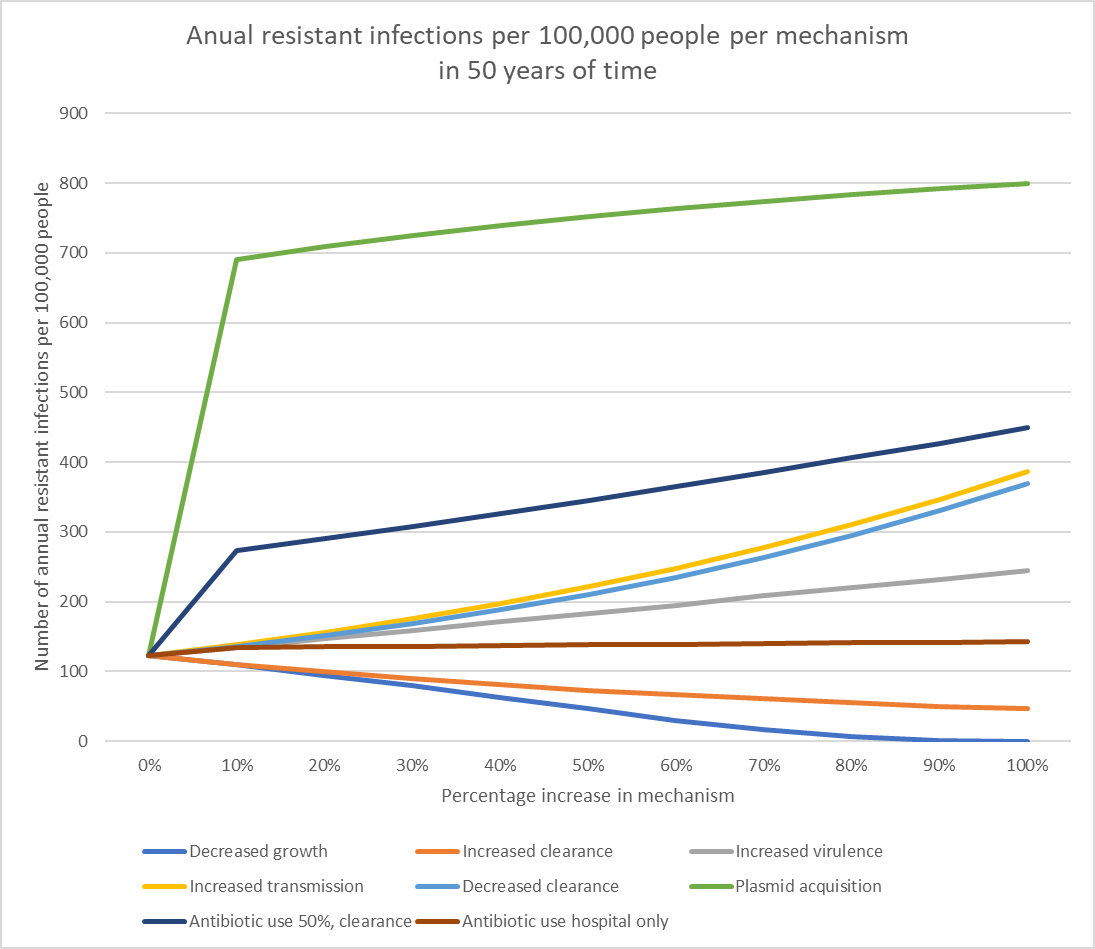
*

S3 Fig. Annual ESBL *E. coli* infections per 100,000 people per mechanism in in 50 years of time

Supplement: S3 Fig — (DOCX) [file pcbi.1009875.s008.docx]

**
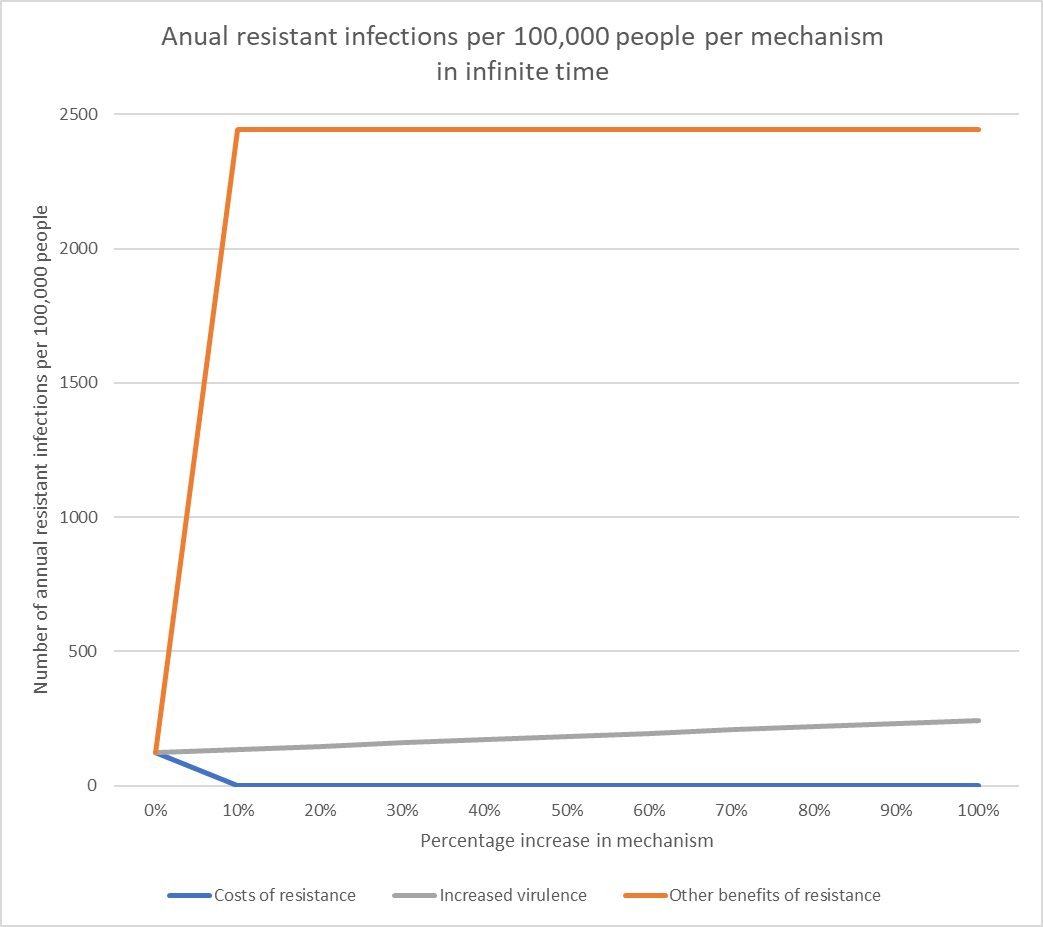
**

S4 Fig. Annual ESBL E. coli infections per 100,000 people per mechanism when letting time run to infinity

Supplement: S4 Fig — (DOCX) [file pcbi.1009875.s009.docx]
