# Supplementary material for: Does plasmid-based beta-lactam resistance increase E. coli infections: Modelling addition and replacement mechanisms
Source: PLoS Comput Biol. 2022 Mar 14;18(3):e1009875. doi: 10.1371/journal.pcbi.1009875 (PMC8947615; doi:10.1371/journal.pcbi.1009875)
Supplement: S5 Fig — (DOCX) [file pcbi.1009875.s010.docx]

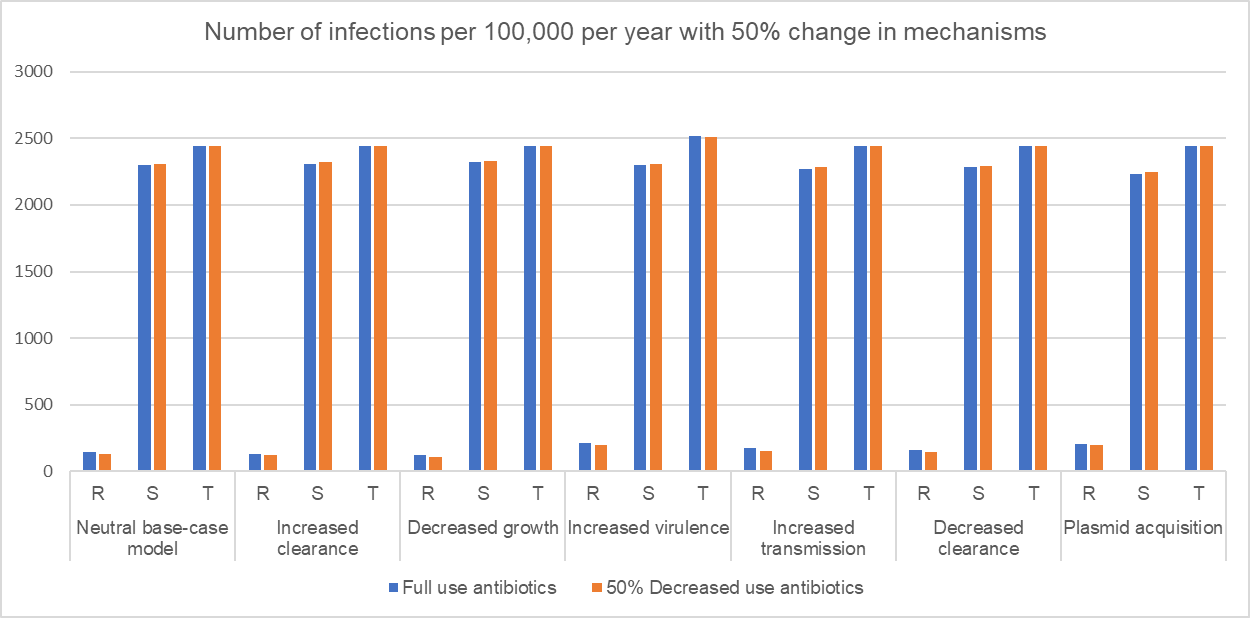


Fig S5. Annual ESBL *E. coli* infections per 100,000 people with 50% change in mechanisms and decreased antibiotics use
