# Supplementary material for: Does plasmid-based beta-lactam resistance increase E. coli infections: Modelling addition and replacement mechanisms
Source: PLoS Comput Biol. 2022 Mar 14;18(3):e1009875. doi: 10.1371/journal.pcbi.1009875 (PMC8947615; doi:10.1371/journal.pcbi.1009875)
Supplement: S1 Table — (DOCX) [file pcbi.1009875.s012.docx]

S1 Table. Annual prevalence of ESBL in E. coli bacteraemia in 24 Dutch hospitals

| Year | Tested samples | ESBL | Prevalence of ESBL (%) | 95% CI |
| --- | --- | --- | --- | --- |
| 2014 | 2675 | 144 | 5.38 | 4·59 – 6·31 |
| 2015 | 2730 | 147 | 5.38 | 4·60 – 6·30 |
| 2016 | 2800 | 166 | 5.93 | 5·11 – 6·87 |
| 2017 | 2842 | 153 | 5.38 | 4·61 – 6·28 |
| 2018 | 3037 | 168 | 5.53 | 4·77 – 6·40 |

*ESBL =* extended spectrum beta-lactamases producing, *CI = confidence interval*
