# Supplementary material for: Does plasmid-based beta-lactam resistance increase E. coli infections: Modelling addition and replacement mechanisms
Source: PLoS Comput Biol. 2022 Mar 14;18(3):e1009875. doi: 10.1371/journal.pcbi.1009875 (PMC8947615; doi:10.1371/journal.pcbi.1009875)
Supplement: S2 Table — (DOCX) [file pcbi.1009875.s013.docx]

S2 Table. Scenarios and mechanisms studied in this paper

|  |  |  | Rate per day | | |
| --- | --- | --- | --- | --- | --- |
| Scenario | Mechanism | Adjusted path | Hospitalized patients | Former patients | Community |
| Fitness cost | Increased clearance | Increases RR to R & SR to S | 0.0028^b^-0.0056 | 0.0028^b^-0.0056 | 0.0028^b^-0.0056 |
|  | Decreased growth | Decreases R to RR | 0.0357^b^-0.00357 | 0.0357^b^-0.00357 | 0.0357^b^-0.00357 |
| Benefit | Increased virulence | Increases probability resistant infections | 0.0106^b^-0.212 | 0.0053^b^-0.0106 | 1.15^10^-5b^-2.30^10^-5^ |
|  | Increased transmission | Increases S to SR & R to RR | 0.0078^b^-0.0156 | 0.0053^b^-0.0106 | 0.00195^b^-0.0039 |
|  | Decreased clearance | Decreases RR to R & SR to S | 0.0028^b^-0.00028 | 0.0028^b^-0.00028 | 0.0028^b^-0.00028 |
|  | Plasmid transfer | Creates path SR to RR | 0·010^b^-0·02 | 0·010^b^-0·02 | 0·010^b^-0·02 |
|  | Antibiotic use | Increases SS to S & SR to R | 0.064^b^-0.128 | 0.032^b^– 0.065 | 0.001-0.002 |
|  | Hospital antibiotic use | Increases SS to S & SR to R | 0.34^b^-0.68 | 0^b^ | 0^b^ |
| Mixed scenario | 20% increased hospital transmission | Increases S to SR & R to RR | 0.0094 |  |  |
|  | 20% increased clearance in community | Increases RR to R & SR to S | 0.0034 |  |  |
| Double benefit scenario | 20% increased virulence | Inc. probability resistant infections | 0.127 | 0.064 | 1.38^10^-5^ |
|  | 20% increased transmission | Increases S to SR & R to RR | 0.0094 | 0.0064 | 0.00234 |
| ^a^ Each mechanism is also shows visually in Fig X. in the mean text  ^b^ Starting value, the same value as for the susceptible variant | | | | | |
