# Supplementary material for: Does plasmid-based beta-lactam resistance increase E. coli infections: Modelling addition and replacement mechanisms
Source: PLoS Comput Biol. 2022 Mar 14;18(3):e1009875. doi: 10.1371/journal.pcbi.1009875 (PMC8947615; doi:10.1371/journal.pcbi.1009875)
Supplement: S3 Table — (DOCX) [file pcbi.1009875.s014.docx]

**S3 Table. In 50 years, the number of resistant and susceptible infections per 100,000 inhabitants under different scenarios**

| Infections | Neutral | Mixed | Double benefit |
| --- | --- | --- | --- |
| Resistant (ARB) | 122 | 100 | 702 |
| Susceptible (non-ARB) | 2320 | 2343 | 1857 |
| Total | 2443 | 2443 | 2560 |
