# Supplementary material for: Does plasmid-based beta-lactam resistance increase E. coli infections: Modelling addition and replacement mechanisms
Source: PLoS Comput Biol. 2022 Mar 14;18(3):e1009875. doi: 10.1371/journal.pcbi.1009875 (PMC8947615; doi:10.1371/journal.pcbi.1009875)
Supplement: S4 Table — (DOCX) [file pcbi.1009875.s015.docx]

**S4 Table. Parameters used in the model**

|  |  | Hospital | Former patients | Community |
| --- | --- | --- | --- | --- |
| Parameter^1^ | Symbol | _h_^2^ | _f_^2^ | _c_^2^ |
| Number of people in compartment^3^ | y |  |  |  |
| Population size | N | 177 | 694 | 99129 |
| Transmission | β | 0·0078[1] | 0·0053[2] | 0·00195 (β_h_ / 4) |
| Clearance rate | λ | 0·0028[3] | 0·0028[3] | 0·0028[3] |
| Infection rate | π | 0·0106[4] | 0·0053 (π_h_ /2) | 1·14874*10^-5^ (Supplementary text 2, own calculations on data of de Greeff & Mouton [5] |
| Admission rate | α |  |  | 0·00020991 (Adjusted from Cooper et al. [6] based on Dutch data. Supplementary text 3) |
| Readmission rate | v |  | 0·0018991 (Adjusted from Cooper et al. [6] based on Dutch data. Supplementary text 3) |  |
| Discharge rate | μ | 0·125[6] |  |  |
| Rate to community | γ |  | 0·03[6] |  |
| Rate of growing to high density | ω | 0·0357[7] | 0·0357[7] | 0·0357[7] |
| Proportion receiving antibiotics* | ε |  | 0 |  |
| Proportion cleared of susceptible strain due to antibiotics* | φ | 0.5 | 0.5 | 0.5 |
| Plasmid transfer* | ϑ | 0·01 | 0·01 | 0·01 |
| External plasmid transfer* | ϑ_2_ | 0·001 | 0·001 | 0·001 |

^*^ Value is 0 in the neutral model
^1^ For each rate, subscripts _s_ (susceptible), _ss_ (high density susceptible), _sr_ (resistant and susceptible), _r_ (resistant) and _rr_ (high density resistant) indicated the rate attributable to those with that specific colonisation state.
^2^ Subscripts _h_ (hospital), _f_ (former patient) and _c_ (community) are used to indicate each population.

^3^ For example, y_rrh_ are hospital patients who are high density colonised with resistant strains, or in other words, hospital patients in compartment RR.

**References**

1. Gurieva T, Dautzenberg MJD, Gniadkowski M, Derde LPG, Bonten MJM, Bootsma MCJ. The Transmissibility of Antibiotic-Resistant Enterobacteriaceae in Intensive Care Units. Clin Infect Dis [Internet]. 2017 Sep 15;66(4):489–93. Available from: https://doi.org/10.1093/cid/cix825

2. Haverkate MR, Platteel TN, Fluit AC, Cohen Stuart JW, Leverstein-van Hall MA, Thijsen SFT, et al. Quantifying within-household transmission of extended-spectrum β-lactamase-producing bacteria. Clin Microbiol Infect [Internet]. 2017 Jan 1 [cited 2019 Nov 15];23(1):46.e1-46.e7. Available from: https://www.sciencedirect.com/science/article/pii/S1198743X16303603

3. Bar-Yoseph H, Hussein K, Braun E, Paul M. Natural history and decolonization strategies for ESBL/carbapenem-resistant Enterobacteriaceae carriage: systematic review and meta-analysis. J Antimicrob Chemother. 2016 Oct;71(10):2729–39.

4. Reddy P, Malczynski M, Obias A, Reiner S, Jin N, Huang J, et al. Screening for extended-spectrum beta-lactamase-producing Enterobacteriaceae among high-risk patients and rates of subsequent bacteremia. Clin Infect Dis. 2007 Oct;45(7):846–52.

5. de Greeff SC, Mouton JW. NethMap 2018: Consumption of antimicrobial agents and antimicrobial resistance among medically important bacteria in the Netherlands / MARAN 2018: Monitoring of Antimicrobial Resistance and Antibiotic Usage in Animals in the Netherlands in 2017 [Internet]. Rijksinstituut voor Volksgezondheid en Milieu RIVM; 2018. Available from: https://rivm.openrepository.com/bitstream/10029/622042/2/2018-0046.pdf

6. Cooper BS, Medley GF, Stone SP, Kibbler CC, Cookson BD, Roberts JA, et al. Methicillin-resistant Staphylococcus aureus in hospitals and the community: stealth dynamics and control catastrophes. Proc Natl Acad Sci U S A. 2004 Jul;101(27):10223–8.

7. Dethlefsen L, Huse S, Sogin ML, Relman DA. The Pervasive Effects of an Antibiotic on the Human Gut Microbiota, as Revealed by Deep 16S rRNA Sequencing. PLOS Biol [Internet]. 2008 Nov 18;6(11):e280. Available from: https://doi.org/10.1371/journal.pbio.0060280
