# Supplementary material for: Does plasmid-based beta-lactam resistance increase E. coli infections: Modelling addition and replacement mechanisms
Source: PLoS Comput Biol. 2022 Mar 14;18(3):e1009875. doi: 10.1371/journal.pcbi.1009875 (PMC8947615; doi:10.1371/journal.pcbi.1009875)
Supplement: S5 Table — (DOCX) [file pcbi.1009875.s016.docx]

**S5 Table. In 10 years, the number of infections per 100,000 people per year for each mechanism**

|  |  | Number of infections per 100,000 per year | | | | | | | | | | |
| --- | --- | --- | --- | --- | --- | --- | --- | --- | --- | --- | --- | --- |
|  |  | Percentage of change in characteristic | | | | | | | | | | |
| Altered characteristic |  | 0 | 10 | 20 | 30 | 40 | 50 | 60 | 70 | 80 | 90 | 100 |
| Increased clearance | R | 122 | 120 | 117 | 115 | 113 | 110 | 108 | 106 | 104 | 103 | 101 |
|  | S | 2320 | 2323 | 2325 | 2328 | 2330 | 2332 | 2334 | 2336 | 2338 | 2340 | 2342 |
|  | T | 2443 | 2443 | 2443 | 2443 | 2443 | 2443 | 2443 | 2443 | 2443 | 2443 | 2443 |
| Decreased growth | R | 122 | 119 | 116 | 111 | 106 | 99 | 91 | 80 | 65 | 43 | 14 |
|  | S | 2320 | 2320 | 2323 | 2327 | 2331 | 2337 | 2343 | 2352 | 2378 | 2399 | 2429 |
|  | T | 2443 | 2443 | 2443 | 2443 | 2443 | 2443 | 2443 | 2443 | 2443 | 2443 | 2443 |
| Increased virulence | R | 122 | 134 | 147 | 159 | 171 | 183 | 195 | 208 | 220 | 232 | 244 |
|  | S | 2320 | 2320 | 2320 | 2320 | 2320 | 2320 | 2320 | 2320 | 2320 | 2320 | 2320 |
|  | T | 2443 | 2455 | 2467 | 2479 | 2491 | 2504 | 2516 | 2528 | 2540 | 2552 | 2565 |
| Increased transmission | R | 122 | 125 | 129 | 132 | 136 | 139 | 143 | 147 | 150 | 154 | 158 |
|  | S | 2320 | 2317 | 2314 | 2311 | 2307 | 2303 | 2300 | 2296 | 2292 | 2288 | 2284 |
|  | T | 2443 | 2443 | 2443 | 2443 | 2443 | 2443 | 2443 | 2443 | 2443 | 2443 | 2443 |
| Decreased clearance | R | 122 | 125 | 127 | 130 | 133 | 136 | 139 | 143 | 146 | 150 | 153 |
|  | S | 2320 | 2318 | 2315 | 2312 | 2309 | 2306 | 2303 | 2300 | 2296 | 2293 | 2289 |
|  | T | 2443 | 2443 | 2443 | 2443 | 2443 | 2443 | 2443 | 2443 | 2443 | 2443 | 2443 |
| Plasmid acquisition | R | 122 | 182 | 183 | 185 | 186 | 187 | 187 | 188 | 189 | 190 | 190 |
|  | S | 2320 | 2261 | 2259 | 2258 | 2257 | 2256 | 2255 | 2254 | 2254 | 2253 | 2252 |
|  | T | 2443 | 2443 | 2443 | 2443 | 2443 | 2443 | 2443 | 2443 | 2443 | 2443 | 2443 |
| Antibiotic use, 50%.clearance | R | 122 | 147 | 149 | 151 | 153 | 155 | 157 | 159 | 162 | 164 | 166 |
|  | S | 2320 | 2296 | 2292 | 2291 | 2289 | 2287 | 2285 | 2283 | 2281 | 2279 | 2277 |
|  | T | 2443 | 2443 | 2443 | 2443 | 2443 | 2443 | 2443 | 2443 | 2443 | 2443 | 2443 |
| Antibiotic use in hospital only | R | 122 | 126 | 126 | 127 | 127 | 127 | 127 | 128 | 128 | 128 | 128 |
|  | S | 2320 | 2317 | 2316 | 2316 | 2316 | 2315 | 2315 | 2315 | 2315 | 2314 | 2314 |
|  | T | 2443 | 2443 | 2443 | 2443 | 2443 | 2443 | 2443 | 2443 | 2443 | 2443 | 2443 |

*R = resistant, S = susceptible, T= total*
