# Supplementary material for: Does plasmid-based beta-lactam resistance increase E. coli infections: Modelling addition and replacement mechanisms
Source: PLoS Comput Biol. 2022 Mar 14;18(3):e1009875. doi: 10.1371/journal.pcbi.1009875 (PMC8947615; doi:10.1371/journal.pcbi.1009875)
Supplement: S6 Table — (DOCX) [file pcbi.1009875.s017.docx]

**S6 Table.** **In 50 years, the number of infections per 100,000 people per year for each mechanism**

|  |  | Number of infections per 100,000 per year | | | | | | | | | | |
| --- | --- | --- | --- | --- | --- | --- | --- | --- | --- | --- | --- | --- |
|  |  | Percentage of change in characteristic | | | | | | | | | | |
| Altered characteristic |  | 0 | 10 | 20 | 30 | 40 | 50 | 60 | 70 | 80 | 90 | 100 |
| Increased clearance | R | 122 | 110 | 99 | 90 | 81 | 73 | 67 | 61 | 55 | 50 | 46 |
|  | S | 2320 | 2333 | 2343 | 2353 | 2361 | 2369 | 2376 | 2382 | 2387 | 2392 | 2397 |
|  | T | 2443 | 2443 | 2443 | 2443 | 2443 | 2443 | 2443 | 2443 | 2443 | 2443 | 2443 |
| Decreased growth | R | 122 | 109 | 94 | 79 | 62 | 46 | 30 | 16 | 6 | 1 | 0 |
|  | S | 2320 | 2334 | 2349 | 2364 | 2380 | 2397 | 2413 | 2427 | 2437 | 2442 | 2443 |
|  | T | 2443 | 2443 | 2443 | 2443 | 2443 | 2443 | 2443 | 2443 | 2443 | 2443 | 2443 |
| Increased virulence | R | 122 | 134 | 147 | 159 | 171 | 183 | 195 | 208 | 220 | 232 | 244 |
|  | S | 2320 | 2320 | 2320 | 2320 | 2320 | 2320 | 2320 | 2320 | 2320 | 2320 | 2320 |
|  | T | 2443 | 2455 | 2467 | 2479 | 2491 | 2504 | 2516 | 2528 | 2540 | 2553 | 2565 |
| Increased transmission | R | 122 | 138 | 155 | 175 | 197 | 221 | 248 | 278 | 311 | 347 | 386 |
|  | S | 2320 | 2305 | 2287 | 2268 | 2246 | 2221 | 2194 | 2164 | 2132 | 2096 | 2056 |
|  | T | 2443 | 2443 | 2443 | 2443 | 2443 | 2443 | 2443 | 2443 | 2443 | 2443 | 2443 |
| Decreased clearance | R | 122 | 136 | 151 | 168 | 188 | 210 | 235 | 263 | 294 | 330 | 369 |
|  | S | 2320 | 2307 | 2291 | 2274 | 2255 | 2233 | 2208 | 2180 | 2148 | 2113 | 2073 |
|  | T | 2443 | 2443 | 2443 | 2443 | 2443 | 2443 | 2443 | 2443 | 2443 | 2443 | 2443 |
| Plasmid acquisition | R | 122 | 691 | 709 | 725 | 739 | 752 | 763 | 774 | 783 | 792 | 800 |
|  | S | 2320 | 1752 | 1734 | 1718 | 1704 | 1691 | 1679 | 1669 | 1659 | 1651 | 1643 |
|  | T | 2443 | 2443 | 2443 | 2443 | 2443 | 2443 | 2443 | 2443 | 2443 | 2443 | 2443 |
| Antibiotic use, 50%.clearance | R | 122 | 273 | 290 | 308 | 326 | 345 | 365 | 385 | 406 | 427 | 450 |
|  | S | 2320 | 2170 | 2153 | 3135 | 2116 | 2097 | 2078 | 2057 | 2036 | 2015 | 1993 |
|  | T | 2443 | 2443 | 2443 | 2443 | 2443 | 2443 | 2443 | 2443 | 2443 | 2443 | 2443 |
| Antibiotic use in hospital only | R | 122 | 134 | 135 | 136 | 137 | 138 | 139 | 140 | 141 | 141 | 142 |
|  | S | 2320 | 2309 | 2308 | 2307 | 2306 | 2305 | 2304 | 2303 | 2302 | 2301 | 2300 |
|  | T | 2443 | 2443 | 2443 | 2443 | 2443 | 2443 | 2443 | 2443 | 2443 | 2443 | 2443 |

*R = resistant, S = susceptible, T= total*
