# Supplementary material for: Does plasmid-based beta-lactam resistance increase E. coli infections: Modelling addition and replacement mechanisms
Source: PLoS Comput Biol. 2022 Mar 14;18(3):e1009875. doi: 10.1371/journal.pcbi.1009875 (PMC8947615; doi:10.1371/journal.pcbi.1009875)
Supplement: S7 Table — (DOCX) [file pcbi.1009875.s018.docx]

**S7 Table.** **Letting time run to infinity, the number of infections per 100,000 people per year for each mechanism**

|  |  | Number of infections per 100,000 per year | | | | | | | | | | |
| --- | --- | --- | --- | --- | --- | --- | --- | --- | --- | --- | --- | --- |
|  |  | Percentage of change in characteristic | | | | | | | | | | |
| Altered characteristic |  | 0 | 10 | 20 | 30 | 40 | 50 | 60 | 70 | 80 | 90 | 100 |
| Increased clearance | R | 122 | 0 | 0 | 0 | 0 | 0 | 0 | 0 | 0 | 0 | 0 |
|  | S | 2320 | 2443 | 2443 | 2443 | 2443 | 2443 | 2443 | 2443 | 2443 | 2443 | 2443 |
|  | T | 2443 | 2443 | 2443 | 2443 | 2443 | 2443 | 2443 | 2443 | 2443 | 2443 | 2443 |
| Decreased growth | R | 122 | 0 | 0 | 0 | 0 | 0 | 0 | 0 | 0 | 0 | 0 |
|  | S | 2320 | 2443 | 2443 | 2443 | 2443 | 2443 | 2443 | 2443 | 2443 | 2443 | 2443 |
|  | T | 2443 | 2443 | 2443 | 2443 | 2443 | 2443 | 2443 | 2443 | 2443 | 2443 | 2443 |
| Increased virulence | R | 122 | 134 | 147 | 159 | 171 | 183 | 195 | 208 | 220 | 232 | 244 |
|  | S | 2320 | 2320 | 2320 | 2320 | 2320 | 2320 | 2320 | 2320 | 2320 | 2320 | 2320 |
|  | T | 2443 | 2455 | 2467 | 2479 | 2491 | 2504 | 2516 | 2528 | 2540 | 2552 | 2565 |
| Increased transmission | R | 122 | 2443 | 2443 | 2443 | 2443 | 2443 | 2443 | 2443 | 2443 | 2443 | 2443 |
|  | S | 2320 | 0 | 0 | 0 | 0 | 0 | 0 | 0 | 0 | 0 | 0 |
|  | T | 2443 | 2443 | 2443 | 2443 | 2443 | 2443 | 2443 | 2443 | 2443 | 2443 | 2443 |
| Decreased clearance | R | 122 | 2443 | 2443 | 2443 | 2443 | 2443 | 2443 | 2443 | 2443 | 2443 | 2443 |
|  | S | 2320 | 0 | 0 | 0 | 0 | 0 | 0 | 0 | 0 | 0 | 0 |
|  | T | 2443 | 2443 | 2443 | 2443 | 2443 | 2443 | 2443 | 2443 | 2443 | 2443 | 2443 |
| Plasmid acquisition | R | 122 | 2443 | 2443 | 2443 | 2443 | 2443 | 2443 | 2443 | 2443 | 2443 | 2443 |
|  | S | 2320 | 0 | 0 | 0 | 0 | 0 | 0 | 0 | 0 | 0 | 0 |
|  | T | 2443 | 2443 | 2443 | 2443 | 2443 | 2443 | 2443 | 2443 | 2443 | 2443 | 2443 |
| Antibiotic use, 50%.clearance | R | 122 | 2443 | 2443 | 2443 | 2443 | 2443 | 2443 | 2443 | 2443 | 2443 | 2443 |
|  | S | 2320 | 0 | 0 | 0 | 0 | 0 | 0 | 0 | 0 | 0 | 0 |
|  | T | 2443 | 2443 | 2443 | 2443 | 2443 | 2443 | 2443 | 2443 | 2443 | 2443 | 2443 |
| Antibiotic use in hospital only | R | 122 | 2443 | 2443 | 2443 | 2443 | 2443 | 2443 | 2443 | 2443 | 2443 | 2443 |
|  | S | 2320 | 0 | 0 | 0 | 0 | 0 | 0 | 0 | 0 | 0 | 0 |
|  | T | 2443 | 2443 | 2443 | 2443 | 2443 | 2443 | 2443 | 2443 | 2443 | 2443 | 2443 |

*R = resistant, S = susceptible, T= total*
