# Supplementary material for: Does plasmid-based beta-lactam resistance increase E. coli infections: Modelling addition and replacement mechanisms
Source: PLoS Comput Biol. 2022 Mar 14;18(3):e1009875. doi: 10.1371/journal.pcbi.1009875 (PMC8947615; doi:10.1371/journal.pcbi.1009875)
Supplement: S8 Table — (DOCX) [file pcbi.1009875.s019.docx]

**S8 Table. Observed growth reported in Ammerlaan et al. [1] and expected growth**

| Observed | 1998 | 2007 |
| --- | --- | --- |
| ARB Enterobacteriaceae | 0·6 | 10·2 |
| ABS Enterobacteriaceae | 22·0 | 35·7 |
| Total | 22·6 | 45·9 |
| Expected |  |  |
| ARB Enterobacteriaceae | 0·6 | 0·97 |
| ABS Enterobacteriaceae | 22·0 | 35·7 |
| Total | 22·6 | 36·67 |

**References**

1. Ammerlaan HSM, Harbarth S, Buiting AGM, Crook DW, Fitzpatrick F, Hanberger H, et al. Secular trends in nosocomial bloodstream infections: antibiotic-resistant bacteria increase the total burden of infection. Clin Infect Dis. 2013 Mar;56(6):798–805.
