# Supplementary material for: Does plasmid-based beta-lactam resistance increase E. coli infections: Modelling addition and replacement mechanisms
Source: PLoS Comput Biol. 2022 Mar 14;18(3):e1009875. doi: 10.1371/journal.pcbi.1009875 (PMC8947615; doi:10.1371/journal.pcbi.1009875)
Supplement: S9 Table — (DOCX) [file pcbi.1009875.s020.docx]

**S9 Table. In 10 years, the number of infections for 50% and 75% change per mechanism when using antibiotics and when decrease overall antibiotic use with 50%**

|  |  |  | Number of infections per 100,000 per year | | | |  | |  |
| --- | --- | --- | --- | --- | --- | --- | --- | --- | --- |
|  |  | | Percentage of change in characteristic | | | | |  | |
|  |  | 50% | |  | 75% | | |  | |
| Altered characteristic |  | Full use antibiotics | Decreased  use antibiotics | Difference (%) | Full use antibiotics | Decreased  use antibiotics | | Difference (%) | |
| Neutral base-case model(no mechanisms change) | R | 145 | 134 | -7.59% |  |  | |  | |
|  | S | 2298 | 2309 | .48% |  |  | |  | |
|  | T | 2443 | 2443 | 0% |  |  | |  | |
| Increased clearance | R | 134 | 122 | -8.96% | 129 | 117 | | -9.30% | |
|  | S | 2309 | 2320 | .48% | 2314 | 2325 | | .48% | |
|  | T | 2443 | 2443 | 0% | 2443 | 2443 | | 0% | |
| Decreased growth | R | 122 | 111 | -9.02% | 95 | 84 | | -11.58% | |
|  | S | 2320 | 2331 | .47% | 2348 | 2358 | | .43% | |
|  | T | 2443 | 2443 | 0% | 2443 | 2443 | | 0% | |
| Increased virulence | R | 217 | 201 | -7.37% | 253 | 234 | | -7.51% | |
|  | S | 2298 | 2309 | .48% | 2298 | 2309 | | .48% | |
|  | T | 2515 | 2509 | -.24% | 2551 | 2543 | | -.31% | |
| Increased transmission | R | 175 | 157 | -10.29% | 191 | 170 | | -10.99% | |
|  | S | 2268 | 2286 | .79% | 2251 | 2273 | | .98% | |
|  | T | 2443 | 2443 | 0% | 2443 | 2443 | | 0% | |
| Decreased clearance | R | 158 | 147 | -6.96% | 166 | 155 | | -6.63% | |
|  | S | 2283 | 2295 | .53% | 2232 | 2287 | | .44% | |
|  | T | 2443 | 2443 | 0% | 2443 | 2443 | | 0% | |
| Plasmid acquisition | R | 208 | 197 | -5.29% | 210 | 199 | | -5.24% | |
|  | S | 2235 | 2245 | .45% | 2277 | 2243 | | -.49% | |
|  | T | 2443 | 2443 | 0% | 2443 | 2443 | | 0% | |

*R = resistant, S = susceptible, T= total*
