# Supplementary material for: Does plasmid-based beta-lactam resistance increase E. coli infections: Modelling addition and replacement mechanisms
Source: PLoS Comput Biol. 2022 Mar 14;18(3):e1009875. doi: 10.1371/journal.pcbi.1009875 (PMC8947615; doi:10.1371/journal.pcbi.1009875)
Supplement: Attachment — Submitted filename: Response Godijk et al.docx [file pcbi.1009875.s022.docx]

Dear Roger Dimitri Kouyos and Virginia Pitzer,

Thank you for the opportunity to revise our manuscript. We like to thank the reviewers for their input and contribution to improve the paper. We value that you really took the time to carefully review our manuscript and your comments are appreciated. We added an acknowledgements section to our manuscript in which we thank the three reviewers for their work. We have thoroughly considered the comments of all three reviewers and addressed them in the point by point response below. We hope that the paper in its current form is suitable for publication in PLOS Computational Biology.

Point-by-point reply:

Reviewer 1.
The authors are investigating with the help of mathematical modelling whether the presence of an antibiotic resistant strain causes an increase in the total number of infections within a population in comparison to a population with only a sensitive strain present or whether the total number of infections is stable and sensitive infections are just replaced by resistant infections. For this investigation different characteristics of resistant strains are considered and their effect. As an exemplary pathogen *E.coli* is examined with and without plasmid-mediated antibiotic resistance and the model is parameterized according to Dutch health care data.

The work covers interesting aspects and shows relevance for a topic of high interest: estimating the burden of antibiotic resistant bacteria. However, I had difficulties with the interpretation of the model and comprehending certain model choices. I think the paper would benefit in some parts with a more detailed explanation and justification of model choices. Generally, it is not an easy task to explain structural neutral epidemiological models, in the following I attach comments, which may be of aid to the authors.

1) As the colonization of the intestinal flora is considered, one patch, I would find the model structure described in equation (14) in the Lipsitch paper easier to interpret biologically as it is hard to see a biological correspondence of the single double colonized compartments if the intestine is not considered as multiple patches (co-infection through infection of multiple patches of one host). Furthermore, in a structure like (14) there would be an interpretable transmission route from the co-colonised compartment to the single colonised compartment.
I am a bit confused by the terminology of “low density colonised” and “high density colonised”, for me this suggests for example that the SR (or SS) compartment has a higher total population of bacteria than the S / R compartment, which should not be the case in a neutral model considering one patch that can be colonized by indistinguishable strains. In L. 285 a maximum colonisation capacity is assumed, which seems to be higher for the (SS, RR, SR) compartments than for S and R. In the simplest case the intestine can be interpreted as one environment, the carrying capacity for *E.coli* should therefore be the same for the double colonised compartments and the singly colonised compartments, except if we assume that the intestine can be seen as two patches. If two patches are assumed in this paper, then further explanation would be helpful.
*Response: Thank you for your comments and suggestion on the colonisation statuses. We understand that we did not clearly explain what we mean with “low density colonised” and “high density colonised” and discussed how to explain this concept better. What we try to explain in our manuscript is that in our model, a high-density colonization corresponds to a stable bacterial composition in which it is hard for other strains to invade. If the bacterial composition is disrupted, e.g., due to disease or antimicrobial treatment, the host can enter the low-colonization state. In this state, strains have the opportunity to invade/outgrow, already present strains and exogeneous acquired strains both have this opportunity. We recognize that previously we did not specify what we mean with maximal colonization capacity, as is the case for the high density colonisation compartments. One page 13, lines 318-320 we added that “As we assume a maximum colonisation capacity, meaning that new strains cannot successfully colonize individuals in state RR, SR or SS , an increased ARB prevalence renders less people susceptible for acquiring new colonisation with susceptible strains”. In the high density colonised compartments an individual is thus indeed no longer susceptible for acquiring a new bacterial strain,
In equation (14) in the Lipsitch paper both strains can be cleared simultaneously, whereas in our manuscript we assume a commensal bacteria, and assume that everyone in the population is colonised with at least one strain.
Importantly, we assume that the daily change to acquire an infection is independent on the state. The states merely determine how vulnerable an individual is for colonization with another strain. If all parameters for susceptible and resistant bacteria are the same, the model does not provide a selective advantage to wither susceptible or resistant bacteria, making the model neutral.*
*When we move away from the neutral null model we start simulating the scenario in which antibiotics are applied, we demonstrate the concept of low and high density colonised. In Figure 5, page 22, providing antibiotics increases the rate from SS to S and SR to R, as the susceptible E. coli are partly cleared by the antibiotics, leaving an unstable and more vulnerable intestinal flora.*

2) If we have two indistinguishable strains (neutral model) shouldn’t the clearance rate be the same for resistant and non-resistant bacteria? (Referring to table S4, where it is described how rates should look like in order to get a neutral model)
*Response: Thank you for this remark and apologies for this unclarity. Table S4 indeed presents the parameters as used in the neutral null model. In the neutral null model, we have clearance for the resistant and susceptible stain which has a rate of 0.0028.
When we test the proposed mechanisms, we move away from the two identical strains, and adjust, under the assumption of the mechanisms increased clearance and decreased clearance, the clearance rate of the resistant strain. In the decreased clearance mechanisms, the clearance of the resistant strain becomes less fast, making it more persistent, and thus giving the resistant strain a benefit (it decreased at most to 0.00028, as can be seen in Table S2). In the example of a mechanisms of increased clearance, the resistant strain is assumed to clear faster than the resistant strain, and has a higher clearance rate (up to 0.0056 as can be seen in Table S2). This gives the resistant strain a disadvantage.
In other words, in the neutral null model, which we start with, the strains are completely identical in their characteristics, e.g., virulence, clearance, growth, transmission rate etc., except for their prevalence. This neutrality allows for the co-existence of two strains with a different prevalence. When we start simulating mechanisms, we move away from the neutrality and characteristics of the stains start to differ. We acknowledge that we did not explicitly state that we move away from the neutral model and added in lines 84-85 “Next we move away from the neutral null model and modify the characteristics of the ARB strain to create benefits or costs of resistance.”*

3) It took me quite a while to understand what is actually assumed by clearance, as not the clearance of infection or colonization is referred to as clearance, but the transition of a double colonised compartments to single colonised compartments.
*Response: Thank you for your comment. We agree with you that we did not explain this in our manuscript, our apologies. We now defined clearance on page 5, lines 95-96 with the sentence “with clearance defined as the rate at which individuals go from high density colonization to low density colonization.”*

Minor comments:
1) L.73 the short form ESBL *E. coli* is used without introducing it before
*Response: Thank you for this remark and apologies for this in accuracy. We changed line 73-75 to “To illustrate this with real observational data, we determined the extended-spectrum beta-lactamase (ESBL) E. coli bacteraemia prevalence in the Netherlands between 2014 and 2019.”*

2) It is the first time where the neutral mathematical model is mentioned (besides the abstract) I think here a reference to a part of the text where a neutral model is explained in more detail would be helpful, as I expect some readers not to be familiar with the concept.
*Response: Thank you for your remark. Indeed, it would be good to refer the that part of the text. We added in line 81-84 the following sentences “In this model, individuals either have a stable, high-density, colonization or a disturbed flora with low-density colonization. The neutral null model is further explained in the base-case scenario in the methods section and the concept of a neutral null model has also been discussed previously (4).”*

3) L. 82/83 would be nice to name an example for the multiple mechanisms
*Response: Thank you for your comment, we agree that this would be good. We added examples in two places. Lines 86-88 now read “Multiple mechanisms, such as increased transmission or antibiotic use, can create a dynamical advantage of resistant over susceptible strains (6,7). To line 90 we added the sentence “An example of a fitness cost could be faster clearance.”*

4) L. 98 definition of cross-transmission?
*Response: Thank you for noticing that we did not explain this, our apologies. We added on lines 108-110 “Cross-transmission is defined here as the transfer of bacteria from one source, in this model another individual, to a receiver which in mechanism five is the subject receiving antibiotics and subsequently having a lower density of susceptible bacteria.” We hope that we now define cross-transmission clearly.*

5) L. 92 reference (6) regarding beta-lactamase-plasmids increasing virulence is cited as a reference on how virulence can be increased, but in the abstract there it is stated that there is no biological evidence that plasmid-based beta-lactam resistance increases virulence. From what the authors describe in the text I understood that the resistance genes itself do not cause an increased virulence. If plasmid-based resistance is considered, can the resistance gene be looked at as a separate unit? Should not the other additional genes which are on the plasmid that cause higher virulence be associated to the resistance?
*Response: Thank you for your comment. You are correct in noting that virulence may also be related to other genes harboured on the ESBL-plasmid. The authors in (6) note that “In addition to antibiotic resistance genes, ESBL-plasmids harbour non-resistance factors, which are partly unexplored. These include fertility and virulence factors…”. We added a sentence on line 100-101 “Moreover, increased virulence may be related to other genes harboured on the ESBL-plasmid (6).” Thank you for your remark.*

6) When introducing the ODES, a reference to table S4 would be helpful to see the definitions of the parameters and the nomenclature/description of compartments.
*Response: Thank you for your comment. We agree that this would be better. We included in lines 263-265 the following sentence: “Table S2 contains the parameters and definitions per mechanisms and Table S4 provides the parameters of transition pathways between the compartments per population.” Moreover, at the end of the section “Base-case scenario” we wrote on line 313 “**The parameters used in the model can be found in Table S4.”. Furthermore, on page 2 of the supplementary material we added “The parameters used in the model can be found in Table S4.”.*

7) Supplement end of the ODE: yr_rro typo?
*Response. Thank you for your comment and a lot of thanks for carefully looking through are equations and supporting information. We changed yr_rro_ to y_rro_.*

8) Maybe I have overseen it, but a mathematical description of the number of people that get infected (infection + cure of infection) and to see where the rate Omega was used would be nice for understanding.
*Response: Thank you for your comment. We added the formulas (16 and 17) for the number of resistant and susceptible infections on lines page 4 of the supporting information. The rate Omega is the rate of growing to high density. It is used in equations 1 to 15. Our apologies, in Table S4 omega was expressed as a capital omega and in the formulas as a small letter omega. In Table S4 we now also write “ω” instead of “Ω”.*

Reviewer 2.

Thank you for the opportunity to read this paper on understanding whether resistant infections in high carriage prevalent commensal bacteria such as *E. coli* emerge in addition to or instead of susceptible infections. The paper offers one of the first (if not the first?) modelling approach to a really difficult question, and I am interested in what it finds.

For me, the modelling presentation could be tightened up that would improve readability and interpretation.

I also have a few queries about the approach and the findings on which I would appreciate the author responses.

1. The testing of the hypotheses is highly conditional on the set up of the model. However, I found the communication of the different models to be very confusing, with a lot of text description and poor connection with the equations given in the Appendix that were not well formatted or particularly well defined. As the model structures are so important to the interpretation of the conclusion of the paper, I would strongly advise the authors to do all their hard work justice by putting together a main text multi-panel compartment diagram that intuitively and quantitatively describes their models (see under Specific comments below for some suggestions).
*Response: Thank you very much for your comments and for taking a close look at our model. We agree with your comments. We added two diagrams, including the different lines, as you suggested to the main text and we added formulas on the number of infections in the supplementary information (as can be seen in the specific comments below). Moreover, we wrote our formulas in Latex to make them more readable.*

2. The research question concerns the incidence of infections, rather than carriage prevalence. It may be a simple point, but it took me a while to understand the set up of the model because everyone in the population is assumed to be colonised, either singly or dually. I would like the authors to be a little more explanatory in their motivation within the main text. That is, the typical resistance data we have characterise the resistance frequencies of infections rather than that of carriage. To observe increased numbers of resistant infections we could imagine them either replacing sensitive infections or adding to the total infections (as per Figs 2-3).

Alternatively, we could imagine an increase in the number of resistant infections because of an increase in the carriage prevalence of resistant strains (again, either instead of or in addition to sensitive strains). Is the second option not supported by data? The authors will know this better than me, but I think that this alternative should be spelled out and reasons given for why this is not consistent with the data we see, or why the model doesn't account for this possibility (as the model structure assumes a 100% carriage prevalence it can't formulate this possibility).	*Response: Thank you very much for your comment. Unlike bacteria such as Staphylococcus aureus, we assume everyone carries E. coli bacteria. We agree that we explain this quite blunt. We expanded our explanation and added the following sentences on page 12, lines 290-295 “E. coli* is a commensal bacterial species but pathogenic variants also exist. *Due to, amongst other ways, point mutations and plasmid transfers E. coli strains can vary in their characteristics. In our model, we assume only two strains, a susceptible and a resistant strain. Carriage with one or two of the strains is possible, but we assume everyone always carries at least one E. coli strain. Hence there is no compartment of uncolonized subjects and individuals can be colonized with resistant strains (R-compartment), susceptible strains (S-compartment) or both (SR-compartment).”
The model presented in this manuscript is currently not suitable for estimating addition and replacement for bacteria that are not carried by everyone, as this would require an uncolonised compartment. We explain this in the discussion, page 10, lines 237-239 with the sentences “In the current analyses the assumption that all individuals carry E. coli is vital. Investigating dynamics of pathogens for which this assumption does not hold, such as Staphylococcus aureus, would require an additional compartment for uncolonised subjects.”
Moreover, previously we indeed did not present our formulas on the infections. However, in the supporting information on page 4, we added formulas 16 and 17 which are used to estimate infections. Figures S2-S4 in the supplementary material, page 10-12 also show the number of infections.
Regarding your note on the option of an increase in the number of resistant infections because of an increase in the carriage prevalence of resistant strains, we agree that this should be clear in the text, and we previously did not stress this explicitly. We added in the discussion section, page 11, lines 239-244 the sentences “Moreover, as we assume that everyone is colonised with E. coli, a mere increase in prevalence of the resistant strain, assuming that characteristics leading to infection are equal to the susceptible strain, does not increase the total number of infections. An increase in prevalence of the resistant strain may cause an increase in the total number of infections for bacteria which are not carried by almost all individuals, as those uncolonised are not at risk for infection and become at risk once colonised.”.*

3. Following from 2. I am confused about the way the different models / hypotheses are tested. The outcome is the number of infections per strain type across the total population as a function of the parameter governing the mechanism. Is this the best way to test the different models? As far as i understand, for each hypothesis, the size of the parameter is varied between 0 and 50% increase away from the null model (i.e. increasing the virulence from 0 to 50% within the "Virulence" model leads to associated increasing the total numbers of all infections). However, is the dialling up and down of the different parameters what would happen when we observe changing numbers of infections? I don't think so. In reality, there will be a fixed parameter value governing a particular mechanism (e.g. a relative virulence of res vs sens strains with a parameter of 10% will give rise to the number of infections we see). The pertinent question is then - under a mechanism - if we implement interventions that, say, reduce antibiotic use, would any resulting reductions in the number of resistant infections be replaced by a compensatory number of sensitive infections. I think to properly address the issue, Fig 3 would show the number of infections of different types as, e.g., antibiotic use decreases, rather than changing a parameter (e.g. pi) that would in reality be fixed.
*Response: Thank you for your comment. You are right in that the scenario which combines a characteristic that differs between the resistant and susceptible strain with an intervention is very interesting to study. We created Table S9 which shows for a 50% and 70% difference per characteristic between the resistant and susceptible strain the number of infections when using antibiotics, and when decreasing antibiotic use with 50%. This allows insight into under which circumstances of changed characteristics of the resistant strain decreasing antibiotic use is a useful intervention. We also included, on page 8, lines 169-173, the following sentences “Finally, we investigated the effect of decreasing antibiotic use by 50% in all three populations on the number of infections under the conditions that each 1) mechanisms was changed by 50% and 2) by 75%. The largest decrease in resistant infections was observed for increased transmission, decreased growth, and increased clearance. Under all conditions, decreased antibiotics use decreased the number of resistant infections. The results can be found in the Table in S9 Table.”*

4. Finally, following on from 3. I found the results unsurprising given the set up of the model. As far as I understand, the total number of infections is determined exclusively by the virulence rate of each of the demographic groups. None of the models alter the number of people in each demographic compartment, and none alter the virulence rate directly (with the exception of the "Virulence" model). Therefore, there is no mechanism through which any of the models (except for Virulence) could change the total number of infections. I found this result a bit limiting. If the authors had comprehensive conceptual diagrams of how the different models (perhaps as suggested below in the specific comments) I think this main limitation would be clearer.
*Response: Thank you for your comment. We completely agree with your suggestions here and we believe that they made our manuscript a lot clearer. We tried to explain this more intuitively in the discussion section, page 8, lines 182-186 with the sentences “This can be explained by increased virulence being the only mechanism which increases the infection rate. The other mechanisms influence parameters affecting colonisation, and subsequently affect the ratio at which resistant and susceptible infections occur. A difference in the ratio of colonisation with susceptible and resistant strains will lead to an equal number of total infections if the infection rate for resistant and susceptible strains is assumed to be equal, because we start with a neutral null model.”. Moreover, we added the diagrams as you suggested (as will be discussed in the specific comments below) and also created Fig. 5 which shows which pathways are affected by the different mechanisms. We will also discuss this figure in the comments below, and it is also included for convenience at the end of this document.*
Some specific suggestions on these points and other comments are given below:
1. The introduction contains many paragraphs of description and justification of the approach. My suggestion would be to give a brief summary of the overall goal of the paper and the method to tackle the goal. The rest can be moved to the Results section by way of a motivation for the Results as the Methods are presented later.
*Response: Thank you for your comment. We understand your suggestion. However, to make the results section readable we decided to provide the information in the sequence in which we presented it. We presented quite elaborate information on the mechanisms, how they are defined and the neutral null model as we felt like the results were otherwise not easy to interpret after reading the introduction. We again would like you to thank you for your suggestion and understand your point of view, but regarding readability and understandability of the work, we decided not to change the information presented in the introduction.*

2. 'Open population' - might be better described consistently as the 'community' or 'community population'? (At least for the differential equations it would be nice to see dc/dt rather than do/dt.)
*Response: Thank you for your comment. We changed open population to community throughout the text and adjusted the formulas accordingly.*

3. I appreciated the description of the models as adjustments away from the neutral 'null' model. I found this a particularly helpful exposition.
*Response: Thank you very much for this compliment.*

4. I found the description of the model particularly confusing as a result of the equations and a repeated but less detailed compartment diagram being in the Supp Mat, with only a text description in the main ms and a simple compartment diagram. My suggestion would be two-fold: first, to add a much more detailed model compartment diagram to the main text; for example, in Colijn et al. (JRSI 2010) the authors use different line types to differentiate between (super)colonisation, clearance, and treatment; further, to create full diagrams that show the different demographic groups and infection compartments, as well as the different model structures (trying to understand Table S3 was a bit of a headache for me). Second, to reformat the equations so they are easier to read. Can anyone in the group perhaps write them in latex? Or at least, not use all the '*' notations and terms like 'sizeo', which makes the terms hard to distinguish and a headache to read.
*Response: Thank you for this suggestion. We adjusted multiple aspects of the manuscript after reading this comment. Firstly, we made a graphical representation of Table S2, namely Fig 5 on page 22 in the main text, which shows which paths are adjusted per mechanism. For convenience, we also added the figure at the end of this document. We added to lines 321-322 on page 14 in the methods section “The influence of each mechanism on the paths in the compartmental model is shown visually in Fig 5.” With this figure we hope that it is easier for readers to grasp how the mechanisms work and how they change the model parameters.
Moreover, we created a new compartmental model as Fig. 4 on page 21 in the main text which has different line types showing transmission, clearance, the rate of growing to high density and clearance due to antibiotic use. In this graph, we also show the different demographic groups and the admission, discharge, readmission and rate of going to community. For convenience, we also added this figure to the end of this document.
Furthermore, we wrote our formulas in Latex as you suggested, they can be seen on pages 1-4 of the supporting information. We deleted all the * as suggested and changed sizeh, sizef and sizec to Nh, Nf and Nc to enhance readability.*

5. Although parameters are defined in Table S4, there seem to be lots of notation that is not defined. For example, do the equations track the fraction of number of infections? What is 'y'. I couldn't properly assess the model because it was all rather difficult to piece together.
*Response: Thank you for your comment. We apologies for making it difficult to assess the model. We added to Table S4 that y is merely the number of people in the compartment. For example, y_rrh_ is the number of patients in the hospital that are high density colonised with resistant strains, thus in compartment rr in the hospital population. To clarify further what is meant with y, we also added an example as a note to the table reading “^3^ For example, y_rrh_ are hospital patients who are high density colonised with resistant strains, or in other words, hospital patients in compartment RR.”. Therefore, the equations calculate the changes in the number of people colonised per time step. We previously did not include the formulas calculating the number of infections, but we did now, on page 4 of the supporting information. Moreover, we changed sizec, as you suggested in your comment above, and we also added a description of this in Table S4.*

6. Some of the model description I find a bit confusing. For example, I don't know what is meant by the following sentences:
a) "In the SR-compartment, the infection rate is the average of the S- and R-compartment, with infections with resistant or susceptible strains being proportional to the two infection rates."
*Response: Thank you for your comments. We will answer your questions per remark. With this sentence we mean that infections do not occur at the rate resistant infections occur, nor at the rate at which susceptible infections occur. Infections in the SR-compartment occur at the infection-rate which is the average of the infection rate of the S- and R-compartment. We indeed explained this to bluntly and apologize for this. With the second part of the sentence “, with infections with resistant or susceptible strains being proportional to the two infection rates” we mean that S and R infections occur proportionally to the infection rate of S and R. As people in the SR compartment can get S as well as R-infections, we had to specify the rate at which these infections occur. We added on lines 308-312 “Infections in the SR-compartment occur at an infection rate which is the average of the infection rate of the S- and R-compartment.* *The proportion of the infections in the SR-compartment, which are caused by susceptible bacteria and by resistant bacteria is proportional to the infection rate in the S- and the R-compartment, as can be seen in formula 16 and 17, page 4 in Supplementary text 1”.*

b) "Without these compartments, introduction of ARB into a population in which everyone could acquire ARB would lead to an equilibrium prevalence of 50%, even in absence of selective advantages." Would this be the single dual infection model (ie X, S, R, SR) as described in Colijn et al. JRSI 2010?
*Response: Thank you for your comment. Yes, it would indeed correspond to this model. This model provides an implicit selective advantage to the strain with the lowest prevalence. We added a reference to Colijn et al. (2021) to the manuscript on page 13, line 301.*

c) "Mechanisms benefitting resistant strains and increasing ARB colonisation prevalence render less people susceptible for acquiring new colonisation with susceptible strains, since we assume a maximum colonisation capacity (RR, SR or SS)"
*Response: Our apologies for this unclarity and thank you for your comment. We mean that mechanisms that benefit the resistant strain create an advantage of the resistant strain. Subsequently, the resistant strain will become more prevalent. Because we assume a maximum colonisation capacity which is either RR, SR or SS, an increased prevalence of the resistant strain will cause more people have the resistant strain and subsequently less people can “still get” the susceptible strain. For example, if someone is in state RR, SR or SS, they can no longer get another ‘S’, this is what we mean with maximum colonisation capacity. Thus, if the prevalence of the resistant strain increases, more people will likely be in the RR and SR states, and at that moment can no longer obtain another S strain. In the manuscript we explained this to bluntly and rewrote the part in lines 317-320 to “Mechanisms benefitting resistant strains increase the ARB colonisation prevalence. As we assume a maximum colonisation capacity, meaning that once at colonisation state RR, SR or SS individuals can no longer acquire a new strain, increased ARB prevalence renders less people susceptible for acquiring new colonisation with susceptible strains.”*

7. Table S4: Should 'infection rate hospital' just be 'infection rate' ? There are no units given for this - or any of the other parameters.
*Response: Thank you for your comment. You are completely right, we also spotted this mistake. We corrected, on page 16 of the supporting information “Infection rate hospital” to “Infection rate”.*

8. The differential equations don't seem to be complete. For example, I can't find where any equation where the rate of infection, \pi, which depends on the group (hosp, inpatient or recently hosp), is used. It would be helpful to show compartments called 'infections' (Sens / Res) that show how the number of infections are tracked, especially as this is the main outcome of the paper.
*Response: Thank you for your comment. We completely agree with you. In the supporting information on page 4, we added equation 16 and 17 which calculate the number of resistant and susceptible infections. These formulas also include the population dependent infection rate π.*

9. "Naturally, introducing costs of resistance without benefits of resistance reduces the incidence of infections caused by ARB and eventually leads to extinction". If you are talking about extinction here, I would suggest rephrasing to carriage (rather than infection), and rename as elimination of resistant strains.
*Response: Thank you for your comment. We agree with your suggestions and changed lines 136-137. to “Naturally, introducing costs of resistance without benefits of resistance reduces the carriage of resistant strains and eventually leads to elimination of resistant strains (Fig S4 and Table S7).”.*

Reviewer 3

In this manuscript, the authors developed a compartmental model to simulate the transmission and evolutionary dynamics of the susceptible and resistant strains of *E. coli* in a broad population. Specifically, the authors subdivide the total population into those staying in hospitals, recently discharged, and the open community population. Each subpopulation is then stratified in terms of colonized by susceptible strain, colonized by resistant strain, infected by susceptible strain, infected by resistant strain, and infected by both susceptible and resistant strains. The authors assume that only infected individuals will be responsible for the transmission of both strains. Further, the authors carefully parameterized model parameters based on credible literature data and then simulated their model to investigate seven scenarios with varying model parameters that represent different aspects of the quality of the resistant strain. The major question is which quality of the resistant strain contributed to the past and current trend of *E. coli* infections. The question on “addition and replacement mechanisms” concerns whether or not the prevalence of resistant strain affects the number of annual infections. The authors found via their simulation that the increased virulence of the resistant strain is the only mechanism that increases the total number of infections.

The problem investigated in this manuscript is vital in ecology and epidemiology and is a very meaningful question for mathematical modelers. The authors have developed a reasonable approach to address this issue and interpret their findings with good arguments. However, some assumptions of the model could impact the results of this study, which are listed below.

1. In their main results, the authors assumed no transmission would occur for infected people (i.e., high density colonized people) in compartments SS and SR. However, in reality, if an infected patient is treated under antibiotics, the resistant strain will dominate under selective pressure. Then there could be dynamics from SR to RR and from SS to SR. The authors addressed such concerns in the appendix text on external plasmid transfer. The authors stated that their simulation under this scenario could result in the extinction of the susceptible strain. These outcomes might be attributable to the assumption on parameter about clearance rate, \lambda, which is assumed to be 0.0028 in the neutral model - this means infected people would stay infected for a year. But in reality, infections can naturally cure within days or weeks. Thus, people do not stay in the high density colonized class for a very long time. Another reason for the outcome on susceptible extinction could be how the external plasmid transfer was modeled: it could be a nonlinear process like strain transmissions, with the transmission rates being comparable to the baseline values.

*Response: Thank you for your comment.*

*We think there is a confusion about the interpretation of the high-density states SS, SR and RR. We interpret them as states with a stable bacterial population in which it is hard for other strains to invade and persist. The ‘low-density’ states S and R correspond to transient states, e.g., due to disease or antibiotic use. The parameter lambda corresponds to the rate at which high-density (healthy) individuals move to the low-density state and it does not correspond to the duration of infection. We have rephrased this more clearly in the text.*

*Regarding the first sentence of your comment, we do allow for transmission from people that are highly density colonized. In other words, they can transmit to others, but cannot be colonised themselves with a new strain as they have reached their maximal colonisation capacity or, explained in other words, have a stable intestinal flora. We changed our explanation of the maximal colonisation capacity to better explain what we mean on page 13, lines 301-305 and 318-322.
We agree with you that under selective pressure, the resistant strain would persevere, whereas the susceptible strain may be cleared. We added a new figure, Fig 5. on page 22 that shows how each mechanism influences the pathways of the compartmental model. For convenience, we also added this figure at the end of this document. When using antibiotics, we assume that those in compartment SS move to S, and those in SR move to R. Once in these compartments they are indeed more vulnerable to 1) either grow to high density from R to RR 2) acquire a resistant strain and move from S to SR and R to RR. We agree with your statement, but we model it with 1 step in between, the step from SS to S and SR to R (as also shown in panel g, Fig 5.).
Regarding your comment on the infection time, we agree with you. We apologize for our ambiguity regarding the difference between infection and colonisation. We previously did not include the formulas calculating the number of infections in the manuscript. We included them now in the supporting information, page 4, formula 16 and 17. We do not model a separate compartment for infections, but we assume that a certain fraction of people carrying (in other words colonised with) the resistant or susceptible strain get an infection. We agree that infections can be cleared within days, but E. coli itself, resistant or not, were found in the literature to be able to stay for a year up to four years before clearing. Therefore, we choose this clearance rate for colonisation.
Plasmid transfer would benefit the resistant strain, creating an advantage of the resistant strain above the susceptible strain. As the resistant strain now has an advantage, it will, as we let time run to infinity, out compete the susceptible strain.*

2. There is a linear rate from each colonized class to the infected class, \omega_s, that has not been mentioned in the manuscript. It might be the rate of infection development, and the parameterization was not mentioned.
*Response: Thank you for your comment. We improved our parameterization, which can be found in Tabel S4. The infection rates are indicated by* *π, and differ for the hospital (π_h_), former patient and (π_f_) community population (π_c_) as can be seen in Table S4. The formulas calculating the number of infections are formulas 16 and 17 in the supporting information, page 4. When we examine the mechanisms increased virulence, the infection rate of the resistant strain becomes higher than the resistant strain as can be seen in Table S2.
Moreover, we agree that we could have stated more explicitly that high density colonised is, not the same as having an infection. We added on page 13, lines 304-305 the sentence “We stress that high density colonisation is not the same as having an infection.”. On page 13, lines 306-312 we changed the part on infections to “Infection rates are equal in high- and low-density compartments and proportional to the number of subjects colonised with resistant and susceptible strains. Infections in the SR-compartment occur at an infection rate which is the average of the infection rate of the S- and R-compartment. Halve of those in the SR-compartment develop resistant infections at the resistant infection rate and halve of those in the SR-compartment develop susceptible infections at the susceptible infection rate. as can be seen in formula 16 and 17 on page 4 in Supplementary text 1.” In other words, we assume that a certain fraction of the population has an infection, but this does not influence the other parameters of the model, e.g. they do not become super spreaders.*

3. It is not clear how the model outcomes, annual number of infections, are measured. Is this quantity measured at the steady state of the system? What do the measurement scenarios “in 10 years of time”, “50 years of time”, and “letting time run to infinity” mean? Or do we measure the annual infections at the end of the 10th year, 50th year, and the ultimate steady state?

*Response: Thank you for bringing this to our attention. We indeed did not provide the formulas for the number of infections. We included the formulas (number 16 and 17) on page 4 of the supporting information. We measure the number of infections as a proportion of the people colonised experiencing an infection. The measurement scenarios calculate the annual number of infections after 10 years.* *We added on page 14 lines 341-344 “Scenarios and mechanisms are studied during time periods of 10 years, 50 years and infinite time. The outcome is the annual number of infections after the subsequent time period (Table S5, S6 and S7 and Fig S2, S3 and S4).”*

4. The parameter value about infection rate is hard to estimate in most related studies. The authors estimated this value by literately interpreting it as “the number of infections that happened per day per person.” Unfortunately, the infection parameter in ordinary differential equation models does not capture this exact interpretation. It only represents the intensity of the transmission force and cannot be directly perceived from real data. Usually, people calibrate this parameter by fitting the model to time-series data (such as most of the COVID-19 modeling studies) or calibrating it into a credible range so that the model outcomes (for example, the percentage of annual infections) fall into the known ranges. In this study, time-series data could be hard to obtain, and the model fitting could be tricky (as there are too many parameters). To revise this estimation, the authors could consider the method in the following paper (this is just an example to point out the specific technique!), which the authors used to calibrate reasonable transmission rates.

<https://journals.plos.org/plosone/article?id=10.1371/journal.pone.0153820>

*Response: Thank you for your comment. We did not include the formulas estimating the number of infections in the previous manuscript, but as discussed above, they can now be found on page 4, formula 16 and 17, of the supporting information. We interpret the infection rate as number of infections per day per person and the formulas provide information on the annual number of infections after a certain time period, e.g., 10 or 50 years. As discussed under comment 3, we clarified this in the manuscript as you suggested. Moreover, we added on page 13, lines 307-308 “The infection rate is defined as the number of infections per day per person.”*

*Moreover, it is not our aim to precisely model how many infections occur. The aim of the study is to estimate whether different biological mechanisms cause addition to or replacement of susceptible infections. For these reasons, we decided not to calibrate infection rate with the method you propose.*

5. The results obtained in Fig 3 is very interesting, and it would be helpful if the authors could explain intuitively from their model and assumption on why most other scenarios do not increase the overall infections.

*Response: Thank you for comment and compliment. We agree that we could have explained this more intuitively. Increased virulence is the only mechanism that increases the parameter infection rate, as we define a more virulent strain to cause more infections. The other mechanisms influence parameters that cause the prevalence of the resistant strain to become higher or lower, therefore causing the ratio of resistant to susceptible infections to become higher or lower. However, they do not change the chance of getting an infection once colonised, and therefore they do not increase the total number of infections. This is because we start with a neutral null model in which we assume that the other characteristics, thus also the infection rate, is the same for the resistant and susceptible strain.*

*We added on page 7, lines 141-142 the sentence “Thus, increased virulence leads to an equivalent addition to the total number of infections, as can be seen in Fig 3.” Furthermore, we explained why the other scenarios do not increase the overall infection in the discussion section, page 8 and 9, lines 181-185 with the sentences “This can be explained by increased virulence being the only mechanism which increases the infection rate. The other mechanisms influence parameters affecting colonisation, and subsequently affect the ratio at which resistant and susceptible infections occur. A difference in the ratio of colonisation with susceptible and resistant strains will lead to an equal number of total infections as the infection rate for resistant and susceptible strains is assumed to be equal, because we start with a neutral null model.”*

*Fig 5.* The biological mechanisms shown in the compartmental model

*Fig 4.* Compartmental model of ESBL *E. coli* and *E. coli* colonisation states
