# Supplementary material for: Does plasmid-based beta-lactam resistance increase E. coli infections: Modelling addition and replacement mechanisms
Source: PLoS Comput Biol. 2022 Mar 14;18(3):e1009875. doi: 10.1371/journal.pcbi.1009875 (PMC8947615; doi:10.1371/journal.pcbi.1009875)
Supplement: Attachment — Submitted filename: Reply Godijk et al 10 01 2022.docx [file pcbi.1009875.s023.docx]

Dear Roger Dimitri Kouyos and Virginia Pitzer,

Thank you for the opportunity to revise our manuscript. We like to thank the reviewers for their input and contribution to improve the paper. We value that you really took the time to carefully review our manuscript and your comments are appreciated. We have thoroughly considered the further comments of all three reviewers and addressed them in the point by point response below. We hope that the paper in its current form is suitable for publication in PLOS Computational Biology. If we need to make any further changes, please let us know and we are more than willing to do so.

Point-by-point reply:

Reviewer 1.

1) The authors thoroughly addressed the points suggested by the reviewers, nice work. I will just mention one point that I still stumbled upon while reading:

(LL.290-305): In lines 297-299 it is described, that the compartments SS and RR are added to ensure a neutral model, but it is stated that those compartments do not have an obvious meaning. In lines 310-311 it is stated that those compartments are interpreted as high-density compartments. Isn't therefore a biological meaning assigned to the compartments?( The implicit assumptions of within-host state of disturbed and stable flora).I think this paragraph might benefit from a clearer formulation to avoid confusion. Furthermore I think it could be made clearer that the double compartments (SS,SR,RR) are seen as the default colonisation of a "healthy" human, which only changes to S and R due to disturbances. (Maybe starting out with explaining the double compartments and then the single colonised ones? But this is probably just a matter of taste)
*Response: Thank you for your remark. We agree that this is confusing. We changed lines 297-306 to “To maintain neutrality in absence of selective advantages, we added SS and RR compartments(4) which we* *interpret as states with a high density of colonization. Without these compartments, introduction of ARB into a population in which everyone could acquire ARB would lead to an equilibrium prevalence of 50%, even in absence of selective advantages (23). Therefore, subjects colonised with susceptible (S) or resistant strains (R) remain susceptible to other strains as long as they are “low density colonised”. Once progressed to high density colonised (labelled SS, RR or SR) they are non-susceptible to acquisition.* *We thus interpret the high-density RR, SR and SS-compartments as stable intestinal flora, preventing colonisation with other bacteria. We stress that high density colonisation is not the same as having an infection. We stress that high density colonisation is not the same as having an infection.*

2) (Typo in L 198?: If ARB would not have increaseD?)
*Response: Thank you for you preciseness. We changed the typo. Line 198 – 200 now read “If ARB would not have increased virulence, the possibility of the addition scenario would be excluded as we found that only increased virulence results in additional infections.”*

Reviewer 2.

1) The authors have responded to my comments very well - thank you for the attention to detail. I especially compliment them on rewriting the models equations and drawing a model diagram. This has made the paper much more user-friendly.

My only remaining comment is really the way that the authors responded to my second point. That is, I was suspicious about the method that tested the hypothesis of replacement of resistant infections. I was arguing that the test of whether resistant strains are in addition or instead of sensitive strains should really be in the context of when the system is perturbed - most notably in the case of introducing an intervention (rather than on a change of parameters that in reality, remain fixed). The authors have included a Table (S9) that assess the impact of these interventions on the number of resistant and sensitive infections with increasing antibiotic control.

Perhaps I am missing something here, but the ‘base case’ scenario - i.e. the first column with ‘full antibiotic use’ indicates different numbers of infections for each mechanism. I don’t believe this is correct way to compare the impact of antibiotic control in the presence of each of the mechanisms. All mechanisms should start with the same base case number of infections and any intervention should therefore be easily compared to each other on the basis of deviation away from this base case.

I would also suggest that this is a figure for ease of understanding - but this is up to the authors’ discretion.

*Response: Thank you for your comments. Thank you for the compliment, we really tried to incorporate the comments when we rewrote the formula’s and created the new diagrams, we agree that these changes make the work a lot clearer.
Regarding your comment on Table S9 and the interventions, in order for all mechanisms to start with the same number of infections the parameters for each mechanism would have to be different. For instance, compare the base case scenario with the increased clearance scenario. If there is increased clearance of resistant strains, the number of infections with resistanct strains will be less in the increased clearance scenario (as is indicated in Table S9). To have the same number of infections with the resistant strain and the same number of infections with the susceptible strains for both scenarios, one has to change another parameter as well, e.g., the transmissibility of resistant strains. Hence a comparison between the base case scenario and the increased clearance scenario with the same number of infections effectively compares the base case scenario with an increased clearance + increased transmissibility scenario. This makes a comparison also difficult. Moreover, as the compensating mechanisms (say transmissibility) would have to be changed with a different percentage for each of the scenarios, a comparison on the effect of changes in antibiotic use would be cumbersome. Hence, we choose to alter only the mechanisms of the scenario to see how changes in antibiotic use influence the number of susceptible and resistant infections.
It is not a lot work to alter the table to have for each scenario the same number of infections with resistant and susceptible strains, but with different transmissibility of the resistant strain between the scenarios, however we are of the opinion that it does not improve the interpretation of the table. If you still feel that it would improve the manuscript, please let us know and we are willing to make the changes. We did alter Table S9 in the sense that we added the neutral base case scenario in which the two strains are equal to each other, and then apply the change in antibiotics. For your convenience we added the Table S9 to the end of this reply. We agree that this presents a neutral starting point after which antibiotic in take is changed. In addition, we added Figures S5 and S6 on page 13 of the supplementary material to present the results clearer. For your convenience we also added the figures at the end of this reply. This was a good suggestion of you to improve the manuscript. Thank you for taking the time for the thorough review.*

2) Finally, there is a Typo on line 173.

*Response: Thank you for your comment. We changes lines 172-173 to “Under all conditions, decreased antibiotics use lowered the number of resistant infections. The results can be found in S9 Table, S5 Fig and S6 Fig.”*

Reviewer 3.

1) The authors were able to clarify most of the concerns raised before. I still have reservations about how the infection rate being estimated in the manuscript, but I can understand that estimating the infection rate is not an easy task and could make the revision impossible. So I do not have further questions about this manuscript.

*Response: Thank you for your response. We are happy that we have clarified your concerns. We like to thank you for your thorough review and comments.*

Fig S5. Annual ESBL *E. coli* infections per 100,000 people with 50% change in mechanisms and decreased antibiotics use

Fig S6. Annual ESBL *E. coli* infections per 100,000 people with 75% change in mechanisms and decreased antibiotics use

**Table S9. In 10 years, the number of infections for 50% and 75% change per mechanism when using antibiotics and when decrease overall antibiotic use with 50%**

|  |  |  | Number of infections per 100,000 per year | | | |  | |  |
| --- | --- | --- | --- | --- | --- | --- | --- | --- | --- |
|  |  | | Percentage of change in characteristic | | | | |  | |
|  |  | 50% | |  | 75% | | |  | |
| Altered characteristic |  | Full use antibiotics | Decreased  use antibiotics | Difference (%) | Full use antibiotics | Decreased  use antibiotics | | Difference (%) | |
| Neutral base-case model (no mechanisms change) | R | 145 | 134 | -7.59% |  |  | |  | |
|  | S | 2298 | 2309 | .48% |  |  | |  | |
|  | T | 2443 | 2443 | 0% |  |  | |  | |
| Increased clearance | R | 134 | 122 | -8.96% | 129 | 117 | | -9.30% | |
|  | S | 2309 | 2320 | .48% | 2314 | 2325 | | .48% | |
|  | T | 2443 | 2443 | 0% | 2443 | 2443 | | 0% | |
| Decreased growth | R | 122 | 111 | -9.02% | 95 | 84 | | -11.58% | |
|  | S | 2320 | 2331 | .47% | 2348 | 2358 | | .43% | |
|  | T | 2443 | 2443 | 0% | 2443 | 2443 | | 0% | |
| Increased virulence | R | 217 | 201 | -7.37% | 253 | 234 | | -7.51% | |
|  | S | 2298 | 2309 | .48% | 2298 | 2309 | | .48% | |
|  | T | 2515 | 2509 | -.24% | 2551 | 2543 | | -.31% | |
| Increased transmission | R | 175 | 157 | -10.29% | 191 | 170 | | -10.99% | |
|  | S | 2268 | 2286 | .79% | 2251 | 2273 | | .98% | |
|  | T | 2443 | 2443 | 0% | 2443 | 2443 | | 0% | |
| Decreased clearance | R | 158 | 147 | -6.96% | 166 | 155 | | -6.63% | |
|  | S | 2283 | 2295 | .53% | 2232 | 2287 | | .44% | |
|  | T | 2443 | 2443 | 0% | 2443 | 2443 | | 0% | |
| Plasmid acquisition | R | 208 | 197 | -5.29% | 210 | 199 | | -5.24% | |
|  | S | 2235 | 2245 | .45% | 2277 | 2243 | | -.49% | |
|  | T | 2443 | 2443 | 0% | 2443 | 2443 | | 0% | |

*R = resistant, S = susceptible, T= total*
