# Supplementary material for: Does plasmid-based beta-lactam resistance increase E. coli infections: Modelling addition and replacement mechanisms
Source: PLoS Comput Biol. 2022 Mar 14;18(3):e1009875. doi: 10.1371/journal.pcbi.1009875 (PMC8947615; doi:10.1371/journal.pcbi.1009875)
Supplement: Attachment — Submitted filename: Reviewer Comments on Manuscript titled.pdf [file pcbi.1009875.s021.pdf]

Reviewer Comments on Manuscript titled "Does plasmid-based beta-lactam resistance increase *E. coli* infections: Modelling addition and replacement mechanisms"

In this manuscript, the authors developed a compartmental model to simulate the transmission and evolutionary dynamics of the susceptible and resistant strains of *E. coli* in a broad population. Specifically, the authors subdivide the total population into those staying in hospitals, recently discharged, and the open community population. Each subpopulation is then stratified in terms of colonized by susceptible strain, colonized by resistant strain, infected by susceptible strain, infected by resistant strain, and infected by both susceptible and resistant strains. The authors assume that only infected individuals will be responsible for the transmission of both strains. Further, the authors carefully parameterized model parameters based on credible literature data and then simulated their model to investigate seven scenarios with varying model parameters that represent different aspects of the quality of the resistant strain. The major question is which quality of the resistant strain contributed to the past and current trend of *E. coli* infections. The question on "addition and replacement mechanisms" concerns whether or not the prevalence of resistant strain affects the number of annual infections. The authors found via their simulation that the increased virulence of the resistant strain is the only mechanism that increases the total number of infections.

The problem investigated in this manuscript is vital in ecology and epidemiology and is a very meaningful question for mathematical modelers. The authors have developed a reasonable approach to address this issue and interpret their findings with good arguments. However, some assumptions of the model could impact the results of this study, which are listed below.

1. In their main results, the authors assumed no transmission would occur for infected people (i.e., high density colonized people) in compartments SS and SR. However, in reality, if an infected patient is treated under antibiotics, the resistant strain will dominate under selective pressure. Then there could be dynamics from SR to RR and from SS to SR. The authors addressed such concerns in the appendix text on external plasmid transfer. The authors stated that their simulation under this scenario could result in the extinction of the susceptible strain. These outcomes might be attributable to the assumption on parameter about clearance rate, \lambda, which is assumed to be 0.0028 in the neutral model - this means infected people would stay infected for a year. But in reality, infections can naturally cure within days or weeks. Thus people do not stay in the high density colonized class for a very long time. Another reason for the outcome on susceptible extinction could be how the external plasmid transfer was modeled: it could be a nonlinear process like strain transmissions, with the transmission rates being comparable to the baseline values.

2. There is a linear rate from each colonized class to the infected class, \omega_s, that has not been mentioned in the manuscript. It might be the rate of infection development, and the parameterization was not mentioned.

3. It is not clear how the model outcomes, annual number of infections, are measured. Is this quantity measured at the steady state of the system? What do the measurement scenarios "in 10 years of time", "50 years of time", and "letting time run to infinity" mean? Or do

we measure the annual infections at the end of the 10th year, 50th year, and the ultimate steady state?

4. The parameter value about infection rate is hard to estimate in most related studies. The authors estimated this value by literately interpreting it as “the number of infections that happened per day per person.” Unfortunately, the infection parameter in ordinary differential equation models does not capture this exact interpretation. It only represents the intensity of the transmission force and cannot be directly perceived from real data. Usually, people calibrate this parameter by fitting the model to time-series data (such as most of the COVID-19 modeling studies) or calibrating it into a credible range so that the model outcomes (for example, the percentage of annual infections) fall into the known ranges. In this study, time-series data could be hard to obtain, and the model fitting could be tricky (as there are too many parameters). To revise this estimation, the authors could consider the method in the following paper (this is just an example to point out the specific technique!), which the authors used to calibrate reasonable transmission rates. https://journals.plos.org/plosone/article?id=10.1371/journal.pone.0153820

5. The results obtained in Fig 3 is very interesting, and it would be helpful if the authors could explain intuitively from their model and assumption on why most other scenarios do not increase the overall infections.
